# Oxygen-Free Csp^3^-H Oxidation of Pyridin-2-yl-methanes to Pyridin-2-yl-methanones with Water by Copper Catalysis

**DOI:** 10.3390/molecules28227587

**Published:** 2023-11-14

**Authors:** Ming Zeng, Jia-Le Chen, Xue Luo, Yan-Jiao Zou, Zhao-Ning Liu, Jun Dai, Deng-Zhao Jiang, Jin-Jing Li

**Affiliations:** 1 School of Pharmacy and Life Science, Jiujiang University, Jiujiang 332005, China; zengming@zjut.edu.cn (M.Z.);; 2 College of Pharmacy, Jiamusi University, Jiamusi 154007, China; m15244643141@163.com (X.L.);; 3 Analytical and Testing Center, Jiujiang University, Jiujiang 332005, China; 4 Jiujiang Key Laboratory for the Development and Utilization of Traditional Chinese Medicine Resources in Northwest Jiangxi, Jiujiang 332005, China

**Keywords:** Csp^3^-H oxidation, pyridin-2-yl-methanones, copper catalysis, water, mechanism study

## Abstract

Aromatic ketones are important pharmaceutical intermediates, especially the pyridin-2-yl-methanone motifs. Thus, synthetic methods for these compounds have gained extensive attention in the last few years. Transition metals catalyze the oxidation of Csp^3^-H for the synthesis of aromatic ketones, which is arresting. Here, we describe an efficient copper-catalyzed synthesis of pyridin-2-yl-methanones from pyridin-2-yl-methanes through a direct Csp^3^-H oxidation approach with water under mild conditions. Pyridin-2-yl-methanes with aromatic rings, such as substituted benzene, thiophene, thiazole, pyridine, and triazine, undergo the reaction well to obtain the corresponding products in moderate to good yields. Several controlled experiments are operated for the mechanism exploration, indicating that water participates in the oxidation process, and it is the single oxygen source in this transformation. The current work provides new insights for water-involving oxidation reactions.

## 1. Introduction

**Scheme 1:**
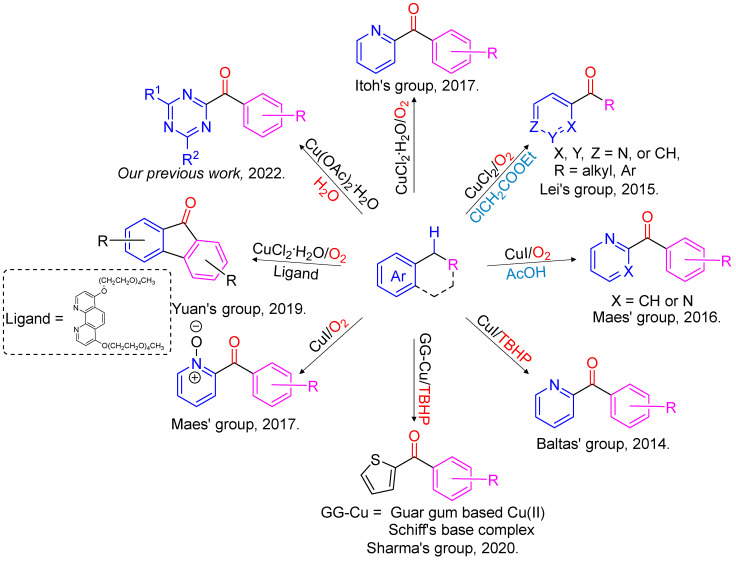
The oxidation of benzylic C(sp^3^)-H bond to aromatic ketones with copper catalysts [20,24,25,28,31,32,33,37].

The synthesis of aromatic ketones has attracted great consideration in recent decades [1,2,3,4,5,6,7,8]. The strategy of direct oxidation of Csp^3^–H provided a powerful and promising method for the transformation of diarylmethane to aromatic ketones. However, an excess of hazardous and dangerous oxidants and a much higher temperature are always introduced due to the low reactivity of C-H bonds [9,10,11,12,13]. As a result, unwanted wastes and by-products are produced, which makes it difficult to obtain desired products in good yields. With the development of organometallic chemistry, the use of transition metals has been investigated in the synthesis of *N*-heterocyclic ketones with molecule oxygen, iodine, and peroxides as oxidants under mild conditions [14,15,16,17,18,19,20,21,22,23,24,25,26,27,28,29,30,31,32,33,34] (Scheme 1). Among all the oxidants, oxygen is more conveniently and readily available. Nevertheless, extra additives, such as NHPI, ClCH_2_COOEt, and AcOH, are essential for some of the examples [24,25,28,29]. According to these reports, peroxy acid intermediate is formed with oxygen via a radical pathway for the transformation, providing an impressive protocol for the synthesis of pyridin-2-yl-methanones. Despite all this, innovative approaches with greener additives by means of metal catalysis are still in great demand. In 2022, Liu has reported the selective oxidation of alkylarenes to aromatic ketones or benzaldehydes with water [35]. In this transformation, water participates in the reaction and offers the oxygen for the process with a palladium catalyst, producing phenyl(pyridin-2-yl)methanone in 44% yield, which inspires us to take water as an oxygen donor for an oxidation reaction in the presence of non-noble metals. More recently, our research group has reported a copper-catalyzed synthesis of aroyl triazines and terminal olefin-substituted triazines [36,37]. Surprisingly, in our attempt to obtain *N*^2^,*N*^2^-dimethyl-*N*^4^-phenyl-6-(1-(pyridin-2-yl)vinyl)-1,3,5-triazine-2,4-diamine, the corresponding oxidation product was observed instead, so we proved that water can provide oxygen for the curtain oxidation transformation. The unexpected findings encourage us to probe the possibility of transforming pyridin-2-yl-methanes to pyridin-2-yl-methanones catalyzed by a copper catalyst in the presence of water. Here, we report an efficient copper-catalyzed synthesis of pyridin-2-yl-methanones via direct Csp^3^-H oxidation with water. To the best of our knowledge, a water-involved oxidation approach for pyridin-2-yl-methanones has never been reported.

## 2. Results and Discussion

We initially conducted the reaction through choosing **1a** as substrate for the optimization study. To our delight, the reaction was smoothly carried out in *N*,*N*-dimethylacetamide (DMA) under a Cu(NO_3_)_2_ **^.^** 3H_2_O/H_2_O/N_2_ catalytic system after 20 h and gave the desired product in 69% yield (Table 1, entry 1). Lowering the amount of water to 2.5 equiv. gave a similar result, but a dramatically decreased yield of **2a** was observed without the use of additional water or anhydrous Cu(NO_3_)_2_ (Table 1, entries 2–4). However, a slightly lower yield was observed in the presence of anhydrous Cu(NO_3_)_2_ and water (Table 1, entry 5). These results suggested that water was essential for the oxidation process. It was worth noting that prolonging the reaction time or elevating the temperature could not help increase the production; contrarily, a shorter reaction time or lower temperature resulted in a decreased yield of **2a** (Table 1, entries 6–9). However, the lower loading of the Cu(NO_3_)_2_ 3H_2_O led to a slower reaction, and a 68% yield of **2a** was obtained when increasing the amount of the catalyst (Table 1, entries 10–11). Next, we paid attention to the various copper (II) catalysts; Cu(NO_3_)_2_ 3H_2_O was proved to be the best choice for this transformation (Table 1, entries 12–16). Finally, the influence of the solvents was investigated (Table 1, entries 17–22). The results showed that the replacement of DMA with DMF, DMSO, or PhCl gave a much lower yield, while the reaction could hardly occur due to the lower solubility in H_2_O or Et_3_N. It is clear that DMA was considered to be optimal for this oxidation process. 

With the optimized condition in hand, a variety of substituted 2-benzylpyridines (**1a-l**) were performed to test the scope of our oxidation methodology. As shown in Table 2, 2-benzylpyridines with electron-donating (-*t*-Bu, -Naphthyl, Ph) and electron-withdrawing (-Cl, -Br, -COMe, -COOMe, -CN, -NO_2_) groups underwent the reaction to afford desired oxidation products in moderate to good yields. Gratifyingly, when 2-(thiophen-2-ylmethyl)pyridine (**1m**), 2-(pyridin-2-ylmethyl)thiazole (**1n**), and 2-(pyridin-3-ylmethyl)pyridine (**1o**) were subjected to the oxidation protocol, the corresponding oxidation products were obtained in 65%, 51%, an 60% yield, respectively. Then, 4-benzylpyridine was tested under the optimized conditions, giving the corresponding product (**2q**) in 62% yield. Despite much effort, 3-benzylpyridine cannot undergo the reaction under the current conditions to form the desired product. Instead, 3-pyridine with triazine substrate (**1r**) could easily transfer into the corresponding product in 66% yield.

**Scheme 2:**
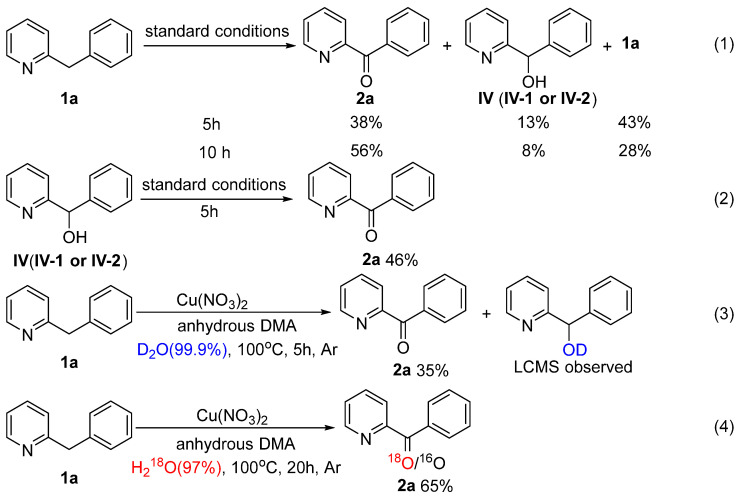
Controlled experiments.

Subsequently, we turned our attention to probe the reaction mechanism of this oxidic process. Firstly, we monitored the reaction mixture over time via liquid chromatography–mass spectrometry (LCMS) for capturing the possible intermediates and by-products, suggesting that **IV** (**IV-1** or **IV-2**) should be a vital intermediate for this transformation (Scheme 2, Equation (1)). A treatment of **IV** (**IV-1** or **IV-2**) under the standard conditions gave the desired product in 46% yield (Scheme 2, Equation (2)). What puzzled us was where the source of oxygen came from. Moreover, several labeling experiments were preformed to investigate the source of oxygen for our oxidation protocol. The reaction was performed in the presence of deuterium oxide or ^18^O-labeled water instead of water (Scheme 2, Equations (3) and (4)); both intermediate **IV** (**IV-1** or **IV-2**) and ^18^O-labeled products were confirmed via LCMS, further proving that water participated in the reaction and acted as oxygen donor in the reaction [37]. 

**Scheme 3:**
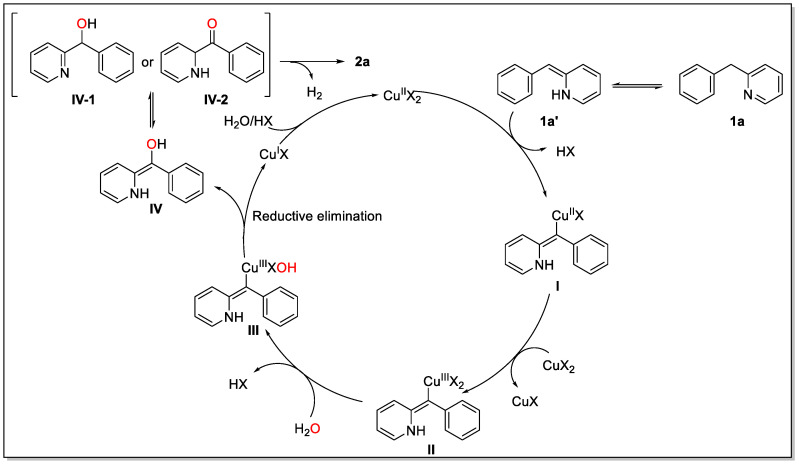
Plausible mechanism.

Based on the results above and previous work [25,37], a plausible mechanism of the water-involved oxidation process was proposed (Scheme 3). Initially, **1a** was activated by a hydrogen proton to give **1a’** [24,25,26,28], which subsequently reacted with CuX_2_ to afford **I** and **II** [33]. Then, the reaction between **II** and H_2_O generating **III** and **IV** (**IV-1** or **IV-2**) was formed through the reductive elimination process [14,36,37,38]. In the presence of a metal catalyst, oxygen, or sodium nitrite [39,40,41,42,43,44,45,46,47,48], **IV** (**IV-1** or **IV-2**) underwent dehydrogenation to afford the desired product **2a [41,46,47,48]** (Scheme 3). Notably, Cu(I) would be reoxidized to Cu(II) in the H_2_/H_2_O/H^+^ system, closing the catalytic cycle [14,37,49].

## 3. Materials and Methods

### 3.1. General Information

Unless otherwise noted, materials were obtained from commercial suppliers and used without further purification. All reactions were performed in a heating mantle in a sealed tube unless otherwise noted. Thin layer chromatography (TLC) was performed using silica gel 60 F254 and was visualized using UV light. Column chromatography was performed with silica gel (mesh 300–400). ^1^H NMR and ^13^C NMR spectra were recorded on a Bruker Avance 400 MHz spectrometer in CDCl_3_ or DMSO-*d_6_* with Me_4_Si as an internal standard. Data were reported as follows: a chemical shift in ppm (*δ*), multiplicity (s = singlet, d = doublet, t = triplet, q = quartet, br = broad, and m = multiplet), coupling constant in Hertz (Hz), and integration. The HRMS and mass data were recorded via ESI on a TOF mass spectrometer.

### 3.2. General Procedure for the Synthesis of ***2***

To a mixture of pyridyl-methanes (1.0 mmol), H_2_O (2.5 mmol), and DMA (3 mL), we added Cu(NO_3_)_2_·3H_2_O (10 mol%). The resulting mixture was then sealed and stirred for 20–40 h at 100 °C under argon. After completion of the reaction, the reaction mixture was cooled to room temperature and extracted with ethyl acetate. The organic phase was dried over anhydrous Na_2_SO_4_. The crude residue was obtained after evaporation of the solvent in a vacuum, and the residue was purified via flash chromatography with petroleum ether and ethyl acetate (*v*/*v* 20/1~5/1) as the eluent to give the pure product.

Phenyl(pyridin-2-yl)methanone (**2a**) [34] ^1^H NMR (400 MHz, CDCl_3_) *δ* 8.77–8.64 (m, 1H), 8.05 (dd, *J* = 8.2, 1.0 Hz, 2H), 8.02 (dd, *J* = 7.9, 0.8 Hz, 1H), 7.88 (td, *J* = 7.7, 1.7 Hz, 1H), 7.61–7.54 (m, 1H), 7.50–7.43 (m, 3H); ^13^C NMR (100 MHz, CDCl_3_) δ 193.9, 155.1, 148.6, 137.0, 136.2, 132.9, 130.9, 128.1, 126.1, 124.6.

(4-(*Tert*-butyl)phenyl)(pyridin-2-yl)methanone (**2b**) [50] ^1^H NMR (400 MHz, CDCl_3_) δ 8.77–8.71 (m, 1H), 8.06–8.01 (m, 3H), 7.91 (td, *J* = 7.7, 1.7 Hz, 1H), 7.57–7.46 (m, 3H), 1.37 (s, 9H); ^13^C NMR (100 MHz, CDCl_3_) δ 193.5, 156.6, 155.4, 148.5, 137.0, 133.5, 130.9, 126.0, 125.2, 124.5, 35.1, 31.1.

Naphthalen-2-yl(pyridin-2-yl)methanone (**2c**) [10] ^1^H NMR (400 MHz, CDCl_3_) δ 8.74–8.69 (m, 1H), 8.30–8.24 (m, 1H), 8.20 (d, *J* = 7.8 Hz, 1H), 8.05 (d, *J* = 8.2 Hz, 1H), 8.00–7.90 (m, 2H), 7.74 (dd, *J* = 7.1, 1.1 Hz, 1H), 7.60–7.49 (m, 4H); ^13^C NMR (100 MHz, CDCl_3_) δ 196.5, 155.5, 149.1, 137.0, 134.7, 133.8, 132.2, 131.2, 129.9, 128.4, 127.4, 126.5, 126.3, 125.6, 124.6, 124.1.

[1,1′-Biphenyl]-4-yl(pyridin-2-yl)methanone (**2d**) [10] ^1^H NMR (400 MHz, CDCl_3_) δ 8.81–8.75 (m, 1H), 8.21–8.16 (m, 2H), 8.11 (d, *J* = 7.8 Hz, 1H), 7.95 (td, *J* = 7.8, 1.7 Hz, 1H), 7.77–7.71 (m, 2H), 7.69–7.64 (m, 2H), 7.54 (dd, *J* = 4.7, 1.2 Hz, 1H), 7.53–7.47 (m, 2H), 7.43 (ddd, *J* = 7.3, 4.7, 1.2 Hz, 1H); ^13^C NMR (100 MHz, CDCl_3_) δ 193.3, 155.2, 148.5, 145.6, 140.1, 137.1, 134.9, 131.6, 128.9, 128.1, 127.3, 126.9, 126.2, 124.6.

Pyridin-2-yl(4-(trifluoromethoxy)phenyl)methanone (**2e**) [51] ^1^H NMR (400 MHz, CDCl_3_) δ 8.73 (dd, *J* = 4.7, 0.6 Hz, 1H), 8.10 (d, *J* = 7.9 Hz, 1H), 8.06 (dt, *J* = 7.7, 1.2 Hz, 1H), 8.02 (br, 1H), 7.93 (td, *J* = 7.7, 1.7 Hz, 1H), 7.56–7.48 (m, 2H), 7.48–7.43 (m, 1H); ^13^C NMR (101 MHz, CDCl_3_) δ 191.1, 148.9, 148.5, 138.1, 137.2, 129.6, 129.5, 126.6, 125.0, 124.7, 124.3, 120.5 (q, *J* = 257.8 Hz); ^19^F NMR (376 MHz, CDCl_3_) δ -57.8.

(4-Chlorophenyl)(pyridin-2-yl)methanone (**2f**) [28] ^1^H NMR (400 MHz, CDCl_3_) δ 8.75 (dd, *J* = 4.4, 0.7 Hz, 1H), 8.13–8.05 (m, 3H), 7.95 (td, *J* = 7.6, 0.7 Hz, 1H), 7.54 (ddd, *J* = 7.6, 4.4, 1.2 Hz, 1H), 7.51–7.46 (m, 2H); ^13^C NMR (100 MHz, CDCl_3_) δ 192.3, 154.6, 148.5, 139.4, 137.2, 134.6, 132.5, 128.4, 126.4, 124.7.

(3-Chlorophenyl)(pyridin-2-yl)methanone (**2g**) [52] ^1^H NMR (400 MHz, CDCl_3_) δ 8.79–8.74 (m, 1H), 8.12–8.07 (m, 2H), 8.00 (dt, *J* = 8.0, 1.1 Hz, 1H), 7.94 (td, *J* = 7.6, 1.7 Hz, 1H), 7.59 (ddd, *J* = 8.0, 2.1, 1.1 Hz, 1H), 7.54 (ddd, *J* = 7.6, 5.0, 1.2 Hz, 1H), 7.45 (t, *J* = 8.0 Hz, 1H); ^13^C NMR (101 MHz, CDCl_3_) δ 192.3, 154.4, 148.6, 137.9, 137.2, 134.3, 132.7, 130.9, 129.5, 129.1, 126.5, 124.7.

(3-Bromophenyl)(pyridin-2-yl)methanone (**2h**) [53] ^1^H NMR (400 MHz, CDCl_3_) δ 8.70 (ddd, *J* = 4.7, 1.5, 0.8 Hz, 1H), 8.18 (dd, *J* = 7.8, 0.7 Hz, 1H), 7.92 (td, *J* = 7.7, 1.7 Hz, 1H), 7.65 (dd, *J* = 7.9, 0.7 Hz, 1H), 7.52–7.48 (m, 1H), 7.49–7.42 (m, 2H), 7.41–7.34 (m, 1H); ^13^C NMR (100 MHz, CDCl_3_) δ 195.8, 153.5, 149.3, 140.3, 137.0, 133.0, 131.5, 129.8, 127.0, 126.9, 123.9, 120.0.

1-(2-Picolinoylphenyl)ethan-1-one (**2i**) [54] ^1^H NMR (400 MHz, CDCl_3_) δ 8.79–8.73 (m, 1H), 8.67 (t, *J* = 1.5 Hz, 1H), 8.31 (dt, *J* = 7.7, 1.3 Hz, 1H), 8.24–8.18 (m, 1H), 8.13 (d, *J* = 7.8 Hz, 1H), 7.96 (td, *J* = 7.7, 1.7 Hz, 1H), 7.62 (t, *J* = 7.8 Hz, 1H), 7.54 (ddd, *J* = 7.6, 4.8, 1.1 Hz, 1H), 2.67 (s, 3H). ^13^C NMR (100MHz, CDCl_3_) δ 197.4, 192.9, 154.4, 148.6, 137.2, 136.9, 136.7, 135.3, 132.1, 130.9, 128.6, 126.6, 124.7, 26.7. 

Methyl 4-picolinoylbenzoate (**2j**) [24] ^1^H NMR (400 MHz, CDCl_3_) δ 8.75 (d, *J* = 4.7 Hz, 1H), 8.21–8.10 (m, 5H), 7.95 (td, *J* = 7.7, 1.7 Hz, 1H), 7.54 (ddd, *J* = 7.7, 4.7, 1.2 Hz, 1H), 3.98 (s, 3H); ^13^C NMR (100 MHz, CDCl_3_) δ 193.2, 166.4, 154.4, 148.6, 139.9, 137.2, 133.5, 130.8, 129.2, 126.6, 124.7, 52.4.

3-Picolinoylbenzonitrile (**2k**) [24] ^1^H NMR (400 MHz, CDCl_3_) δ 8.76 (d, *J* = 4.5 Hz, 1H), 8.50 (t, *J* = 1.4 Hz, 1H), 8.39 (dt, *J* = 7.8, 1.4 Hz, 1H), 8.17 (d, *J* = 7.6 Hz, 1H), 7.98 (td, *J* = 7.6, 1.7 Hz, 1H), 7.88 (dt, *J* = 7.8, 1.4 Hz, 1H), 7.65 (t, *J* = 7.8 Hz, 1H), 7.58 (ddd, *J* = 7.6, 4.5, 1.1 Hz, 1H); ^13^C NMR (100 MHz, CDCl_3_) δ 191.2, 153.7, 148.6, 137.4, 137.2, 135.5, 135.0, 134.9, 129.1, 126.9, 124.8, 118.2, 112.5.

(3-Nitrophenyl)(pyridin-2-yl)methanone (**2l**) [24] ^1^H NMR (400 MHz, CDCl_3_) δ 9.08–8.97 (m, 1H), 8.77 (dd, *J* = 2.7, 2.0 Hz, 1H), 8.57–8.42 (m, 2H), 8.20 (d, *J* = 8.0 Hz, 1H), 7.99 (td, *J* = 7.7, 1.7 Hz, 1H), 7.72 (t, *J* = 8.0 Hz, 1H), 7.59 (ddd, *J* = 7.7, 4.8, 1.1 Hz, 1H); ^13^C NMR (100 MHz, CDCl_3_) δ 191.0, 153.6, 148.7, 147.9, 137.6, 137.4, 136.6, 129.2, 127.0, 126.9, 126.2, 124.8.

Pyridin-2-yl(thiophen-2-yl)methanone (**2m**) [55] ^1^H NMR (400 MHz, CDCl_3_) δ 8.76 (ddd, *J* = 4.7, 1.6, 0.8 Hz, 1H), 8.41 (dd, *J* = 3.9, 1.2 Hz, 1H), 8.19 (dt, *J* = 7.7, 1.2 Hz, 1H), 7.90 (td, *J* = 7.7, 1.6 Hz, 1H), 7.76 (dd, *J* = 5.0, 1.2 Hz, 1H), 7.51 (ddd, *J* = 7.7, 4.7, 1.2 Hz, 1H), 7.20 (dd, *J* = 5.0, 3.9 Hz, 1H); ^13^C NMR (100 MHz, CDCl_3_) δ 183.5, 154.0, 148.2, 140.0, 137.1, 136.7, 136.3, 127.6, 126.6, 123.8.

Pyridin-2-yl(thiazol-2-yl)methanone (**2n**) [56] ^1^H NMR (400 MHz, CDCl_3_) δ 8.85 (d, *J* = 4.5 Hz, 1H), 8.37 (d, *J* = 7.6 Hz, 1H), 8.22 (d, *J* = 3.0 Hz, 1H), 7.96 (td, *J* = 7.6, 1.7 Hz, 1H), 7.80 (d, *J* = 3.0 Hz, 1H), 7.59 (ddd, *J* = 7.6, 4.5, 1.1 Hz, 1H); ^13^C NMR (101 MHz, CDCl_3_) δ 181.5, 161.7, 152.4, 148.8, 144.9, 137.2, 127.5, 127.3, 124.9.

Pyridin-2-yl(pyridin-3-yl)methanone (**2o**) [10] ^1^H NMR (400 MHz, CDCl_3_) δ 9.34 (s, 1H), 8.79 (d, *J* = 3.9 Hz, 1H), 8.73 (d, *J* = 4.3 Hz, 1H), 8.43 (dt, *J* = 7.9, 1.9 Hz, 1H), 8.14 (d, *J* = 7.9 Hz, 1H), 7.93 (td, *J* = 7.7, 1.7 Hz, 1H), 7.53 (ddd, *J* = 7.6, 4.8, 1.1 Hz, 1H), 7.44 (dd, *J* = 7.9, 4.9 Hz, 1H); ^13^C NMR (101 MHz, CDCl_3_) δ 192.0, 153.9, 152.8, 152.1, 148.6, 138.2, 137.2, 132.0, 126.8, 124.5, 123.0.

Phenyl(pyridin-4-yl)methanone (**2p**)[14] ^1^H NMR (400 MHz, CDCl_3_) δ 8.84 (d, *J* = 4.7 Hz, 2H), 7.84 (d, *J* = 7.5 Hz, 2H), 7.67 (t, *J* = 7.5 Hz, 1H), 7.61 (d, *J* = 4.7 Hz, 2H), 7.54 (t, *J* = 7.5 Hz, 2H); ^13^C NMR (101 MHz, CDCl_3_) δ 195.1, 150.3, 144.4, 135.92, 133.5, 130.1, 128.8, 128.6, 122.8.

(4-(Dimethylamino)-6-(phenylamino)-1,3,5-triazin-2-yl)(pyridin-3-yl)methanone (**2r**) ^1^H NMR (400 MHz, DMSO-*d_6_*) δ 10.02 (s, 1H), 9.17 (d, *J* = 1.4 Hz, 1H), 8.86 (dd, *J* = 4.7, 1.4 Hz, 1H), 8.46–8.27 (m, 1H), 7.77 (d, *J* = 7.2 Hz, 2H), 7.61 (dd, *J* = 7.8, 4.7 Hz, 1H), 7.31 (t, *J* = 7.2 Hz, 2H), 7.02 (t, *J* = 7.2 Hz, 1H), 3.20 (s, 3H), 3.12 (s, 3H); ^13^C NMR (100 MHz, DMSO) δ 190.6, 168.5, 164.9, 163.7, 154.5, 151.5, 139.7, 138.1, 130.4, 129.0, 124.3, 123.0, 120.4, 36.6, HRMS (ESI) [M + H]^+^, calcd for C_17_H_17_N_6_O: 321.1464, found: 321.1468.

## 4. Conclusions

In conclusion, we have demonstrated an efficient copper-catalyzed oxygen-free synthesis of pyridin-2-yl-methanones via the direct oxidation of Csp^3^-H with water. Further mechanism studies proved that the oxygen of the products came from water. This work provided a powerful approach for certain oxidation reactions. Detailed mechanistic studies and substrate expansion are in progress.

## Figures and Tables

**Table 1:**
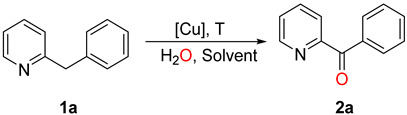
Optimization of the reaction ^a^.

Entry	Cu Salt (mol %)	Water	Solvent	Time/h	Yield/%
1	Cu(NO_3_)_2_·3H_2_O (10 mol%)	5.0 equiv.	DMA	20	69
2	Cu(NO_3_)_2_·3H_2_O (10 mol%)	2.5 equiv.	DMA	20	68
3	Cu(NO_3_)_2_·3H_2_O (10 mol%)	-	DMA	20	36
4	Cu(NO_3_)_2_ (10 mol%)	-	DMA	20	8
5	Cu(NO_3_)_2_ (10 mol%)	2.5 equiv.	DMA	20	59
6	Cu(NO_3_)_2_·3H_2_O (10 mol%)	2.5 equiv.	DMA	20	70 ^b^
7	Cu(NO_3_)_2_·3H_2_O (10 mol%)	2.5 equiv.	DMA	20	43 ^c^
8	Cu(NO_3_)_2_·3H_2_O (10 mol%)	2.5 equiv.	DMA	12	41
9	Cu(NO_3_)_2_·3H_2_O (10 mol%)	2.5 equiv.	DMA	30	61
10	Cu(NO_3_)_2_·3H_2_O (5 mol%)	2.5 equiv.	DMA	20	40
11	Cu(NO_3_)_2_·3H_2_O (20 mol%)	2.5 equiv.	DMA	20	68
12	Cu(OAc)·H_2_O (10 mol%)	2.5 equiv.	DMA	20	42
13	CuCl_2_·2H_2_O (10 mol%)	2.5 equiv.	DMA	20	45
14	CuSO_4_ (10 mol%)	2.5 equiv.	DMA	20	36
15	CuBr_2_ (10 mol%)	2.5 equiv.	DMA	20	40
16	Cu(CF_3_SO_3_)_2_ (10 mol%)	2.5 equiv.	DMA	20	22
17	Cu(NO_3_)_2_·3H_2_O (10 mol%)	2.5 equiv.	DMF	20	45
18	Cu(NO_3_)_2_·3H_2_O (10 mol%)	2.5 equiv.	DMSO	20	30
19	Cu(NO_3_)_2_·3H_2_O (10 mol%)	2.5 equiv.	NMP	20	21
20	Cu(NO_3_)_2_·3H_2_O (10 mol%)	2.5 equiv.	PhCl	20	23
21	Cu(NO_3_)_2_·3H_2_O (10 mol%)	2.5 equiv.	Et_3_N	20	trace
22	Cu(NO_3_)_2_·3H_2_O (10 mol%)	2.5 equiv.	H_2_O	20	trace

^a^ Reaction conditions: **1** (1 mmol), CuX_2_ (10 mol%), solvent (3 mL), H_2_O (2.5 equvi.), 100 °C, 20 h, argon atmosphere. ^b^ 120 °C. ^c^ 80 °C.

**Table 2:**
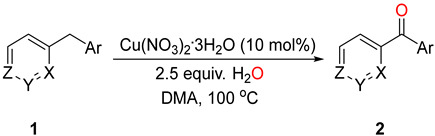
Scope of the Cu catalyzed oxidation of benzylpyridines.

Entry	1	Ar	Time/h	2	Yield/%
1	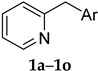 **1a**–**1o**	Ph	20	**2a**	68
2		4-*t*-BuC_6_H_4_	38	**2b**	76
3		2-naphthyl	30	**2c**	85
4		4-PhC_6_H_4_	30	**2d**	92
5		4-OCF_3_C_6_H_4_	25	**2e**	65
6		4-ClC_6_H_4_	20	**2f**	48
7		3-ClC_6_H_4_	20	**2g**	63
8		3-BrC_6_H_4_	30	**2h**	62
9		2-CH_3_COC_6_H_4_	50	**2i**	63
10		4-CH_3_OOCC_6_H_4_	23	**2j**	60
11		3-CNC_6_H_4_	39	**2k**	68
12		3-NO_2_C_6_H_4_	30	**2l**	54
13		2-thiophenyl	25	**2m**	65
14		2-thiazolyl	24	**2n**	51
15		3-pyridyl	20	**2o**	60
16	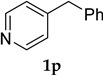 **1p**	-	30	**2p**	62
17	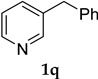 **1q**	-	40	**2q**	trace
18	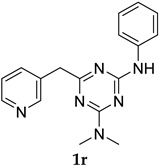 **1r**	-	10	**2r**	66

Reaction conditions: **1** (1 mmol), Cu(NO_3_)_2_**^.^**3H_2_O (10 mol%), solvent (3 mL), H_2_O (2.5 equiv.), 100 °C, argon atmosphere.

## Data Availability

Data are contained within the article and Supplementary Materials.

## Supplementary Materials

The following supporting information can be downloaded at: https://www.mdpi.com/article/10.3390/molecules28227587/s1, Figure S1, ^1^H NMR spectrum of phenyl(pyridin-2-yl)methanone (**2a**); Figure S2, ^13^C NMR spectrum of phenyl(pyridin-2-yl)methanone (**2a**); Figure S3, ^1^H NMR spectrum (4-(tert-butyl)phenyl)(pyridin-2-yl)methanone (**2b**); Figure S4, ^13^C NMR spectrum of (4-(tert-butyl)phenyl)(pyridin-2-yl)methanone (**2b**); Figure S5, ^1^H NMR spectrum of naphthalen-2-yl(pyridin-2-yl)methanone (**2c**); Figure S6, ^13^C NMR spectrum of naphthalen-2-yl(pyridin-2-yl)methanone (**2c**); Figure S7, ^1^H NMR spectrum of [1,1′-biphenyl]-4-yl(pyridin-2-yl)methanone (**2d**); Figure S8, ^13^C NMR spectrum of [1,1′-biphenyl]-4-yl(pyridin-2-yl)methanone (**2d**); Figure S9, ^1^H NMR spectrum of pyridin-2-yl(4-(trifluoromethoxy)phenyl)methanone (**2e**); Figure S10, ^13^C NMR spectrum of pyridin-2-yl(4-(trifluoromethoxy)phenyl)methanone (**2e**); Figure S11, ^19^F NMR spectrum of pyridin-2-yl(4-(trifluoromethoxy)phenyl)methanone (**2e**); Figure S12, ^1^H NMR spectrum of (4-chlorophenyl)(pyridin-2-yl)methanone (**2f**); Figure S13, ^13^C NMR spectrum of (4-chlorophenyl)(pyridin-2-yl)methanone (**2f**); Figure S14, ^1^H NMR spectrum of (3-chlorophenyl)(pyridin-2-yl)methanone (**2g**); Figure S15, ^13^C NMR spectrum of (3-chlorophenyl)(pyridin-2-yl)methanone (**2g**); Figure S16, ^1^H NMR spectrum of (2-bromophenyl)(pyridin-2-yl)methanone (**2h**); Figure S17, ^13^C NMR spectrum of (2-bromophenyl)(pyridin-2-yl)methanone (**2h**); Figure S18, ^1^H NMR spectrum of 1-(2-picolinoylphenyl)ethan-1-one (**2i**); Figure S19, ^13^C NMR spectrum of 1-(2-picolinoylphenyl)ethan-1-one (**2i**); Figure S20, ^1^H NMR spectrum of methyl 4-picolinoylbenzoate (**2j**); Figure S21, ^13^C NMR spectrum of methyl 4-picolinoylbenzoate (**2j**); Figure S22, ^1^H NMR spectrum of 3-picolinoylbenzonitrile (**2k**); Figure S23, ^13^C NMR spectrum of 3-picolinoylbenzonitrile (**2k**); Figure S24, ^1^H NMR spectrum of (3-nitrophenyl)(pyridin-2-yl)methanone (**2l**); Figure S25, ^13^C NMR spectrum of (3-nitrophenyl)(pyridin-2-yl)methanone (**2l**); Figure S26, ^1^H NMR spectrum of pyridin-2-yl(thiophen-2-yl)methanone (**2m**); Figure S27, ^13^C NMR spectrum of pyridin-2-yl(thiophen-2-yl)methanone (**2m**); Figure S28, ^1^H NMR spectrum of pyridin-2-yl(thiazol-2-yl)methanone (**2n**); Figure S29, ^13^C NMR spectrum of pyridin-2-yl(thiazol-2-yl)methanone (**2n**); Figure S30, ^1^H NMR spectrum of pyridin-2-yl(pyridin-3-yl)methanone (**2o**); Figure S31, ^13^C NMR spectrum of pyridin-2-yl(pyridin-3-yl)methanone (**2o**); Figure S32, ^1^H NMR spectrum of phenyl(pyridin-4-yl)methanone (**2q**); Figure S33, ^13^C NMR spectrum of phenyl(pyridin-4-yl)methanone (**2q**); Figure S34, ^1^H NMR spectrum of (4-(dimethylamino)-6-(phenylamino)-1,3,5-triazin-2-yl)(pyridin-3-yl)methanone (**2r**); Figure S35, ^13^C NMR spectrum of (4-(dimethylamino)-6-(phenylamino)-1,3,5-triazin-2-yl)(pyridin-3-yl)methanone (**2r**).
