# Supplementary material for: Oxygen-Free Csp3-H Oxidation of Pyridin-2-yl-methanes to Pyridin-2-yl-methanones with Water by Copper Catalysis
Source: Molecules. 2023 Nov 14;28(22):7587. doi: 10.3390/molecules28227587 (PMC10673412; doi:10.3390/molecules28227587)
Supplement: Supplementary file 1 [file molecules-28-07587-s001.zip › molecules-2686682-supplementary.pdf]

Supporting information

Oxygen Free Csp^3-H Oxidation of Pyridin-2-yl-methanes to Pyridin-2-yl-methanones with Water by Copper Catalysis

Ming Zeng [1], Jia-Le Chen [1], Xue Luo [2], Yan-Jiao Zou [1], Zhao-Ning Liu [2] Jun Dai [3], Deng-Zhao Jiang [1,4] and Jin-Jing Li [2,*]

1 School of Pharmacy and Life Science, Jiujiang University, Jiujiang 332005, China; zengming@zjut.edu.cn (M.Z.)

2 College of Pharmacy, Jiamusi University, Jiujiang 332005, China; m15244643141@163.com (X.L.)

3 Analytical and Testing Center, Jiujiang University, Jiujiang 332005, China

4 Jiujiang Key Laboratory for the Development and Utilization of Traditional Chinese Medicine Resources in Northwest Jiangxi, Jiujiang 332005, China

* Correspondence: lijinjing@jmsu.edu.cn

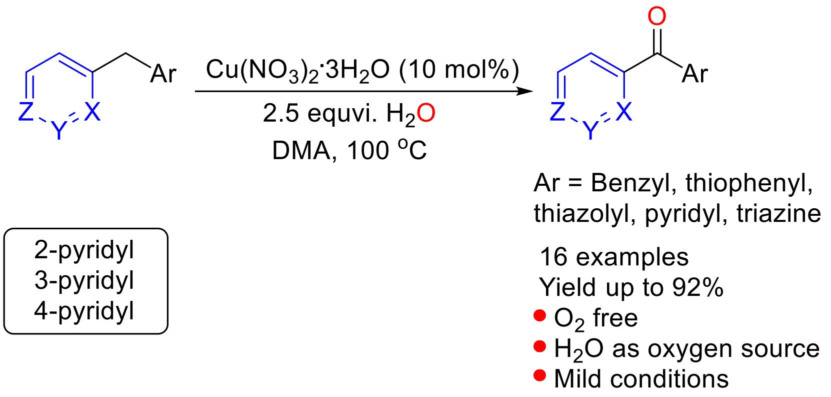

We describe an efficient copper catalyzed synthesis of pyridin-2-yl-methanones from pyridin-2-yl-methanes by direct Csp^3-H oxidation approach with water under mild conditions. Pyridin-2-yl-methanes with aromatic rings such as substituted benzene, thiophene, thiazole, pyridine, triazine, undergo the reaction well to obtain the corresponding products in moderate to good yields. Several controlled experiments are operated for the mechanism exploration, indicating that water participates in the oxidation process and it is the single oxygen source in this transformation. The current work provides a new sight for water involved oxidation reactions.

Contents

Figures	NMR spectra	Pages
1	^{1}H NMR spectra of **2a**	S1
2	^{13}C NMR spectra of **2a**	S2
3	^{1}H NMR spectra of **2b**	S3
4	^{13}C NMR spectra of **2b**	S4
5	^{1}H NMR spectra of **2c**	S5
6	^{13}C NMR spectra of **2c**	S6
7	^{1}H NMR spectra of **2d**	S7
8	^{13}C NMR spectra of **2d**	S8
9	^{1}H NMR spectra of **2e**	S9
10	^{13}C NMR spectra of **2e**	S10
11	^{19}F NMR spectra of **2e**	S11
12	^{1}H NMR spectra of **2f**	S12
13	^{13}C NMR spectra of **2f**	S13
14	^{1}H NMR spectra of **2g**	S14
15	^{13}C NMR spectra of **2g**	S15
16	^{1}H NMR spectra of **2h**	S16
17	^{13}C NMR spectra of **2h**	S17
18	^{1}H NMR spectra of **2i**	S18
19	^{13}C NMR spectra of **2i**	S19
20	^{1}H NMR spectra of **2j**	S20
21	^{13}C NMR spectra of **2j**	S21
22	^{1}H NMR spectra of **2k**	S22
23	^{13}C NMR spectra of **2k**	S23
24	^{1}H NMR spectra of **2l**	S24
25	^{13}C NMR spectra of **2l**	S25
26	^{1}H NMR spectra of **2m**	S26
27	^{13}C NMR spectra of **2m**	S27
28	^{1}H NMR spectra of **2n**	S28
29	^{13}C NMR spectra of **2n**	S29
30	^{1}H NMR spectra of **2o**	S30
31	^{13}C NMR spectra of **2o**	S31
32	^{1}H NMR spectra of **2p**	S32
33	^{13}C NMR spectra of **2p**	S33
34	^{1}H NMR spectra of **2r**	S34
35	^{13}C NMR spectra of **2r**	S35

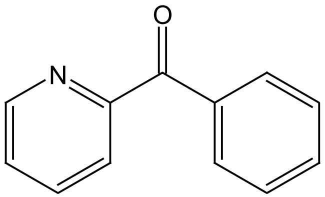

Figure S1. ^{1}H spectrum of **2a** in $CDCl_3$

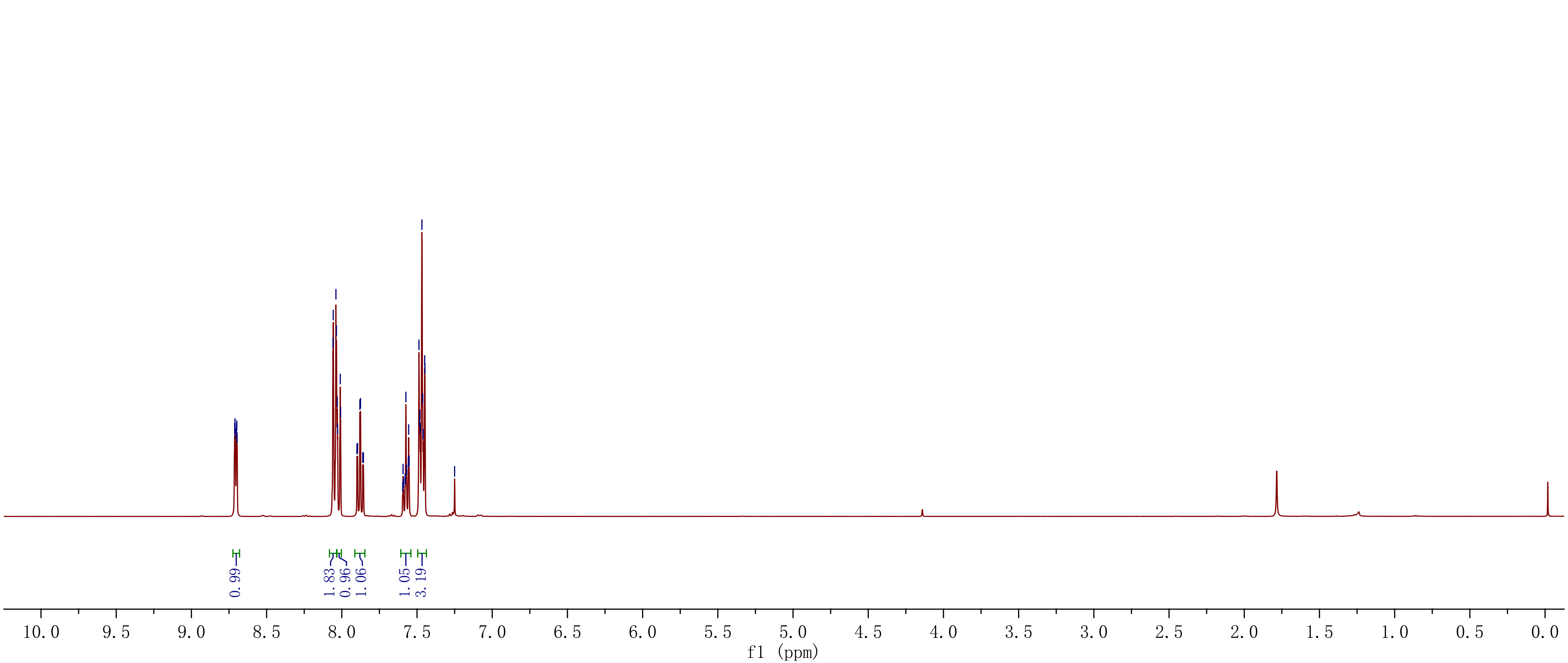

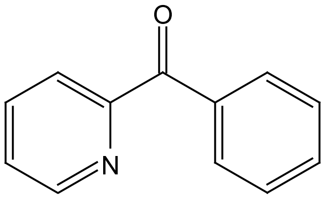

Figure S2. ^{13}C spectrum of **2a** in $CDCl_3$

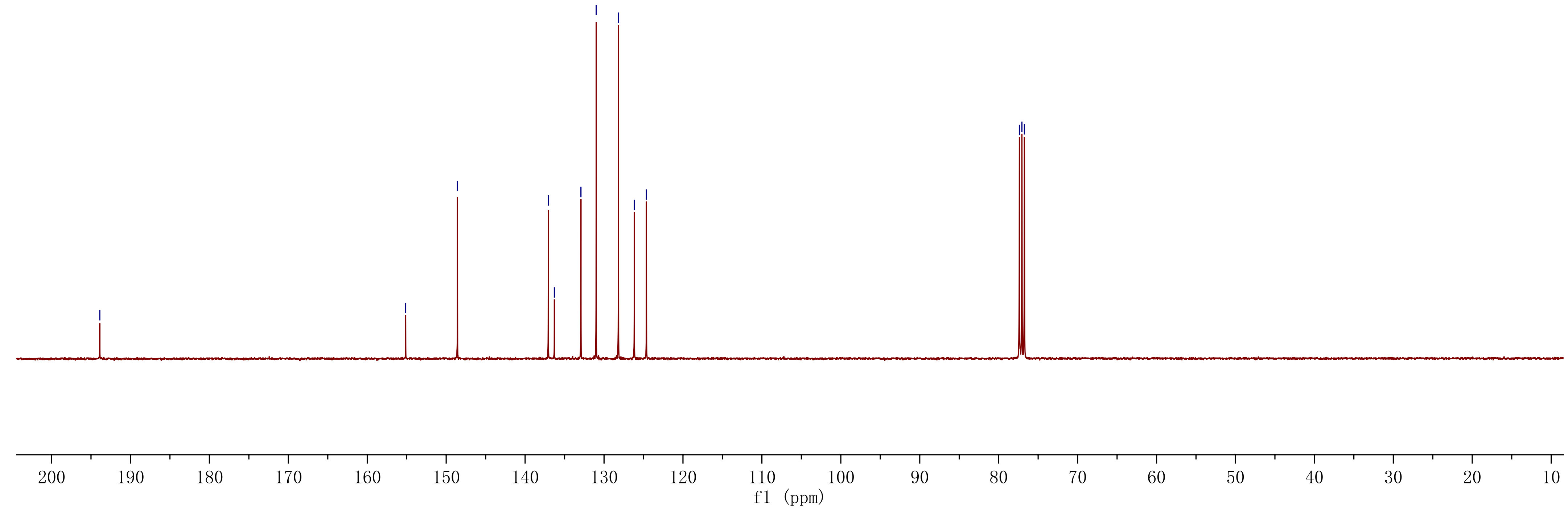

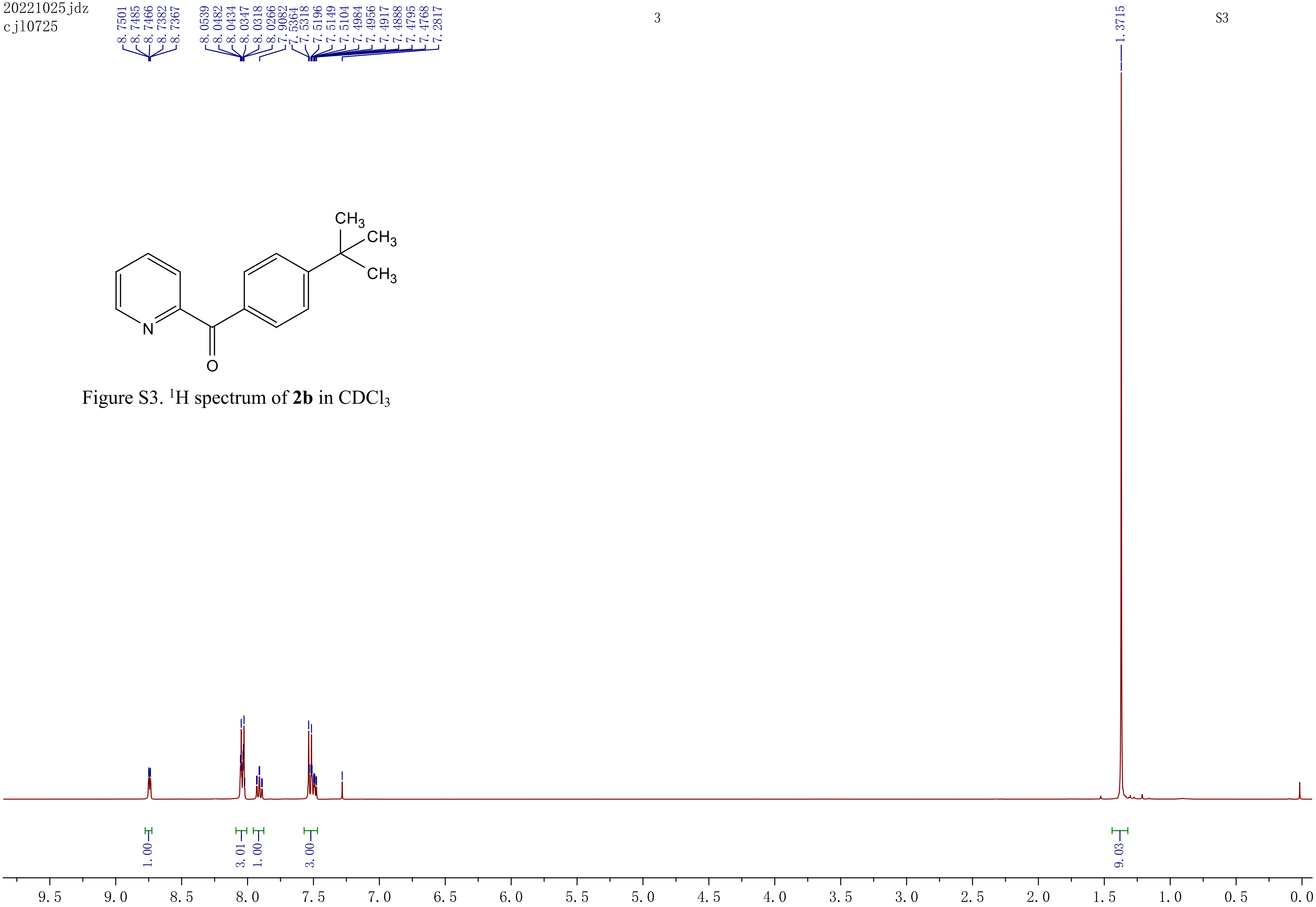

Figure S3. ^{1}H spectrum of **2b** in $CDCl_3$

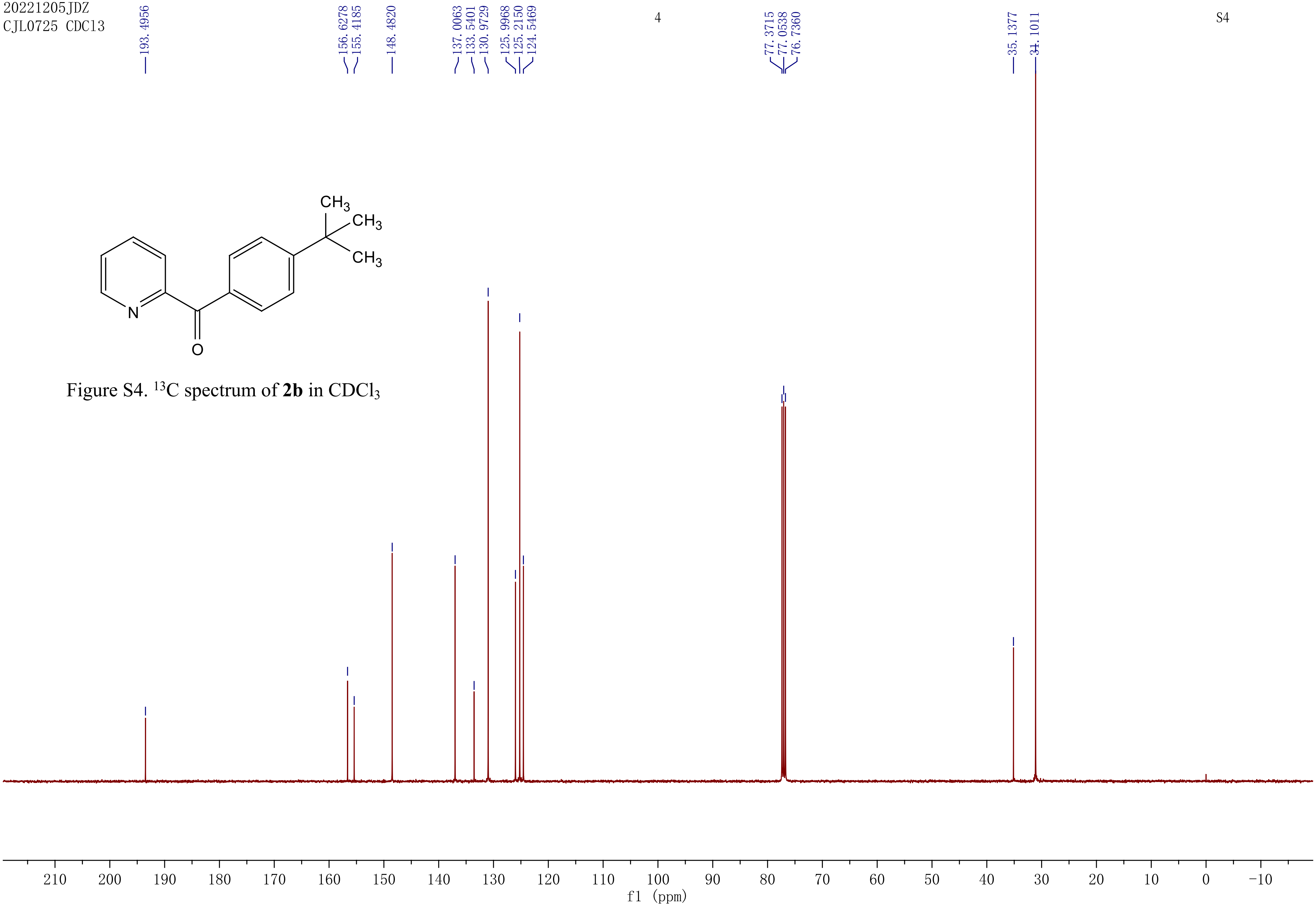

Figure S4. ^{13}C spectrum of **2b** in $CDCl_3$

CJL0323
cjl0323 CDCl3

5

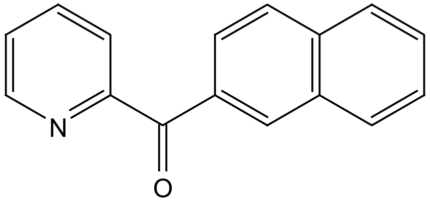

Figure S5. ^{1}H spectrum of **2c** in $CDCl_3$

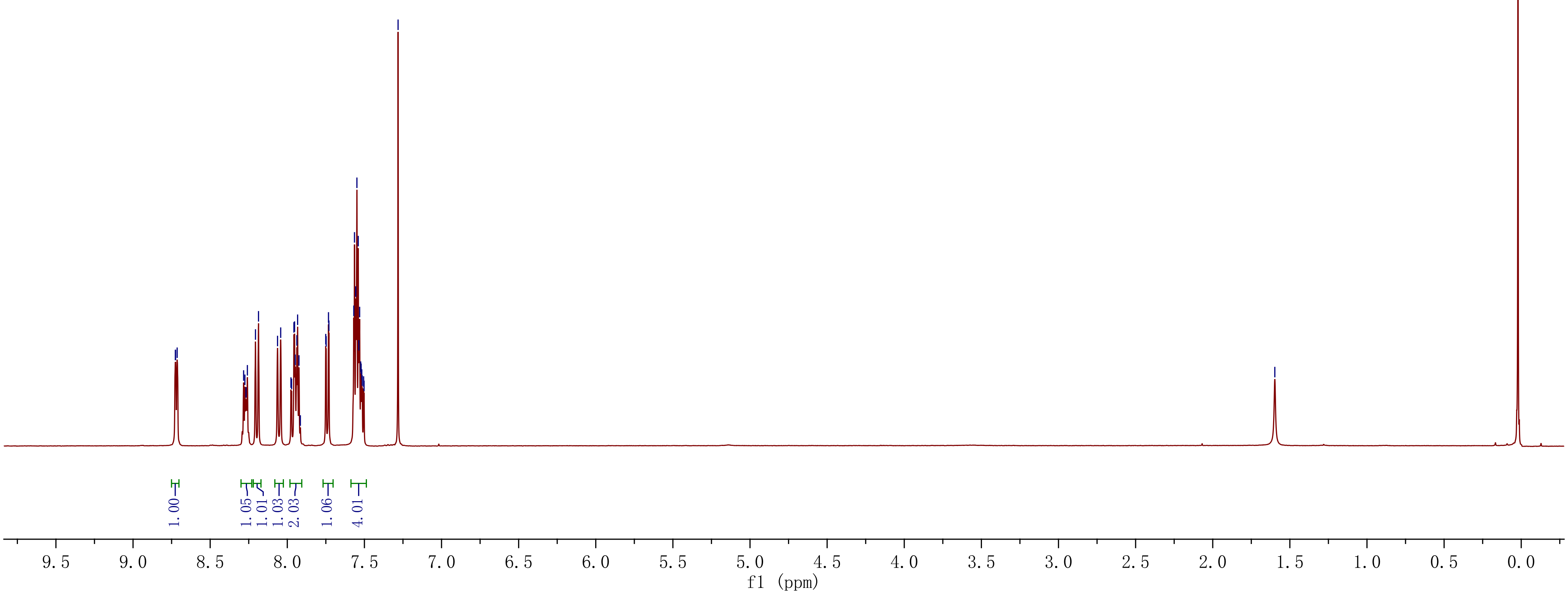

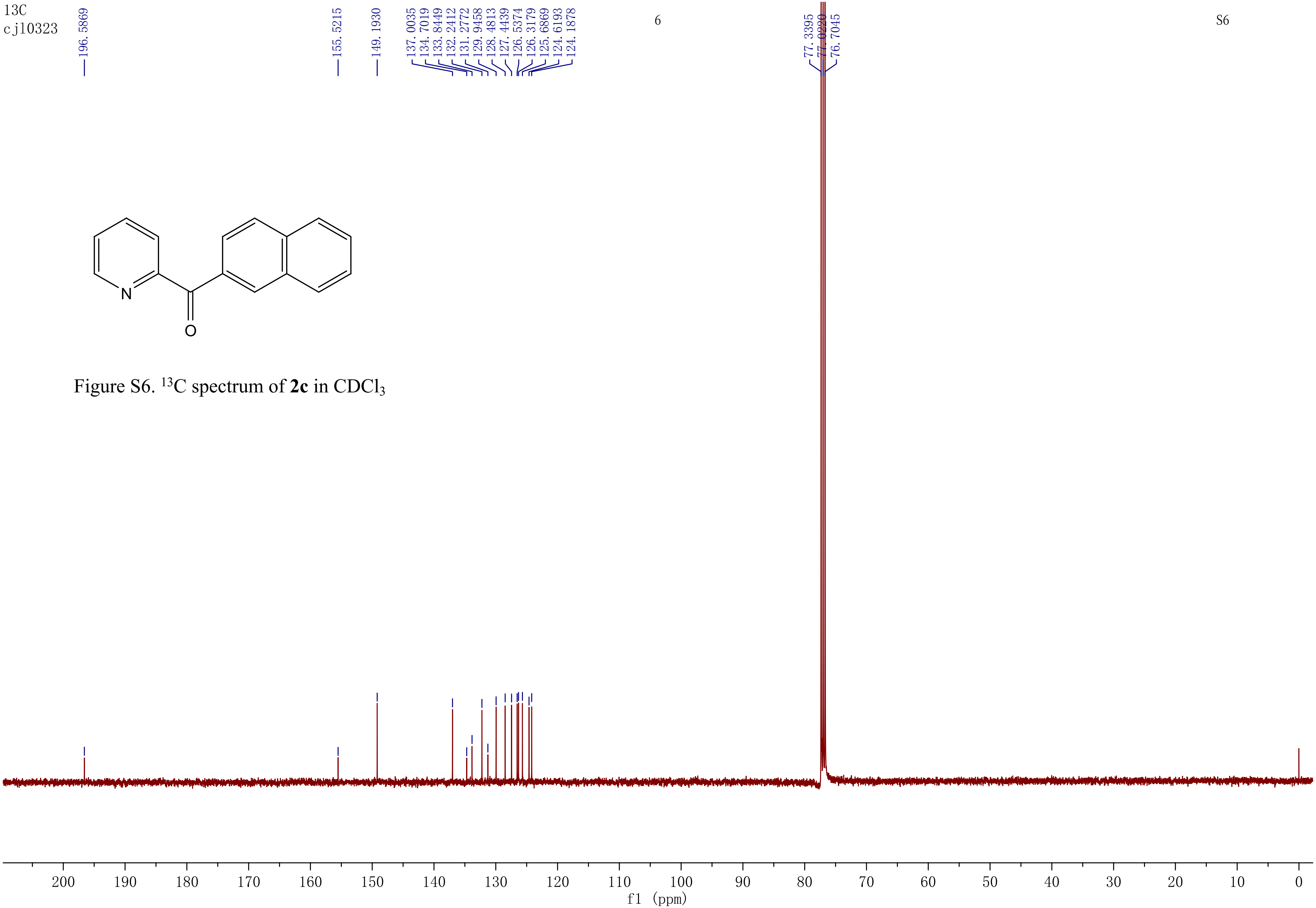

Figure S6. ^{13}C spectrum of **2c** in $CDCl_3$

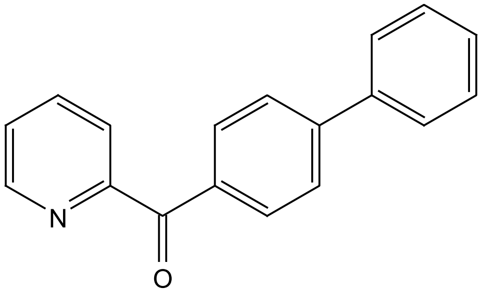

Figure S7. ^{1}H spectrum of **2d** in $CDCl_3$

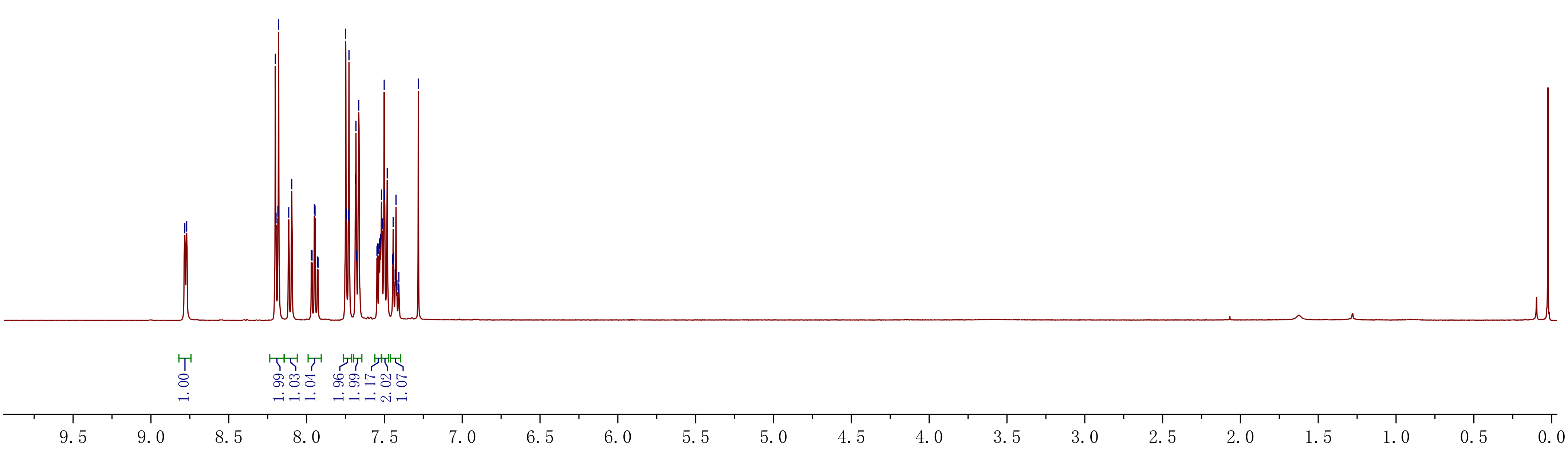

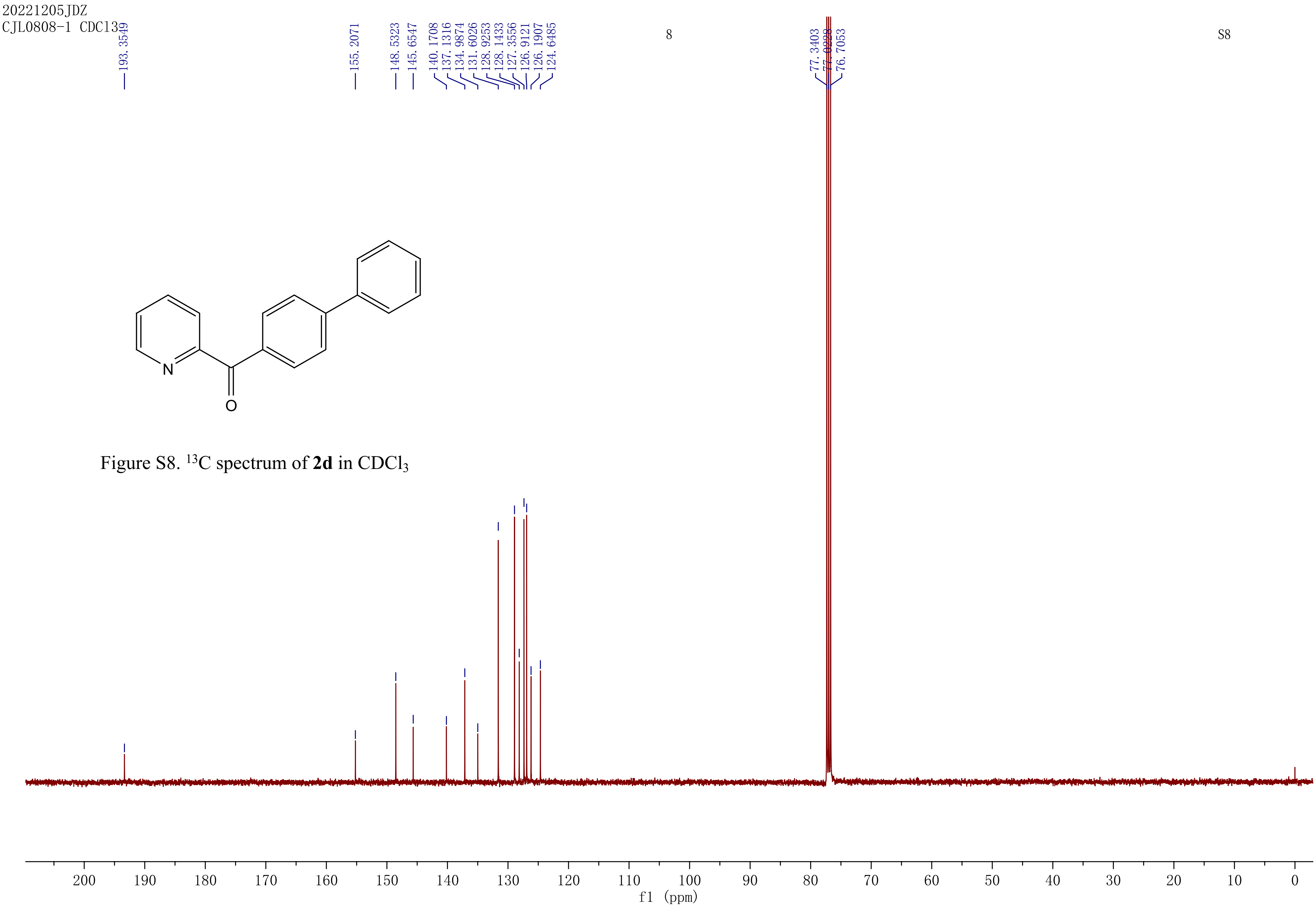

Figure S8. ^{13}C spectrum of **2d** in $CDCl_3$

CJL1125

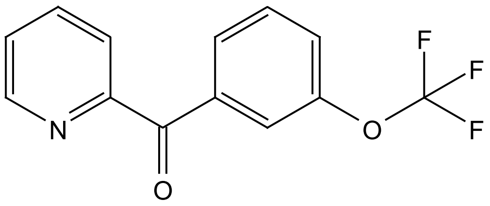

Figure S9. ^{1}H spectrum of **2e** in $CDCl_3$

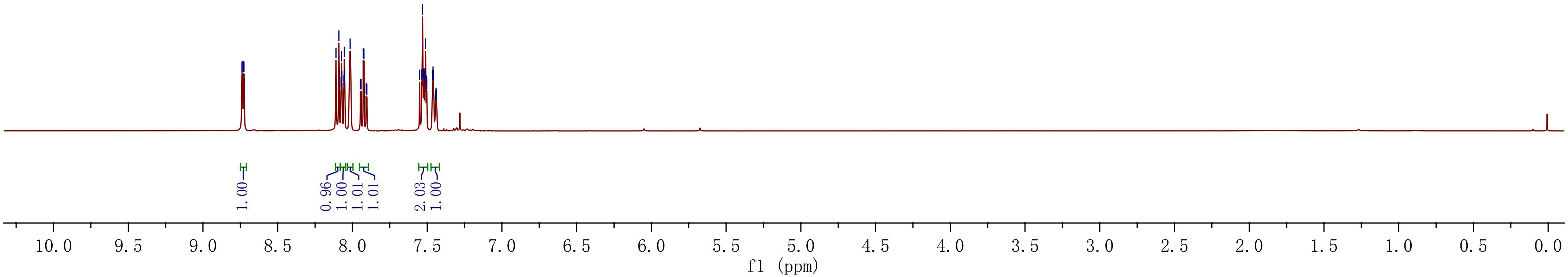

CJL1125
CJL1125

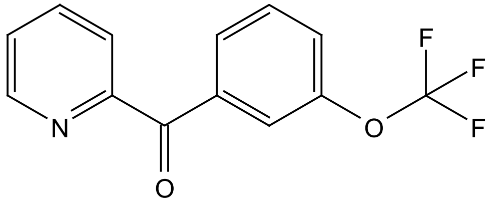

Figure S10. ^{13}C spectrum of **2e** in $CDCl_3$

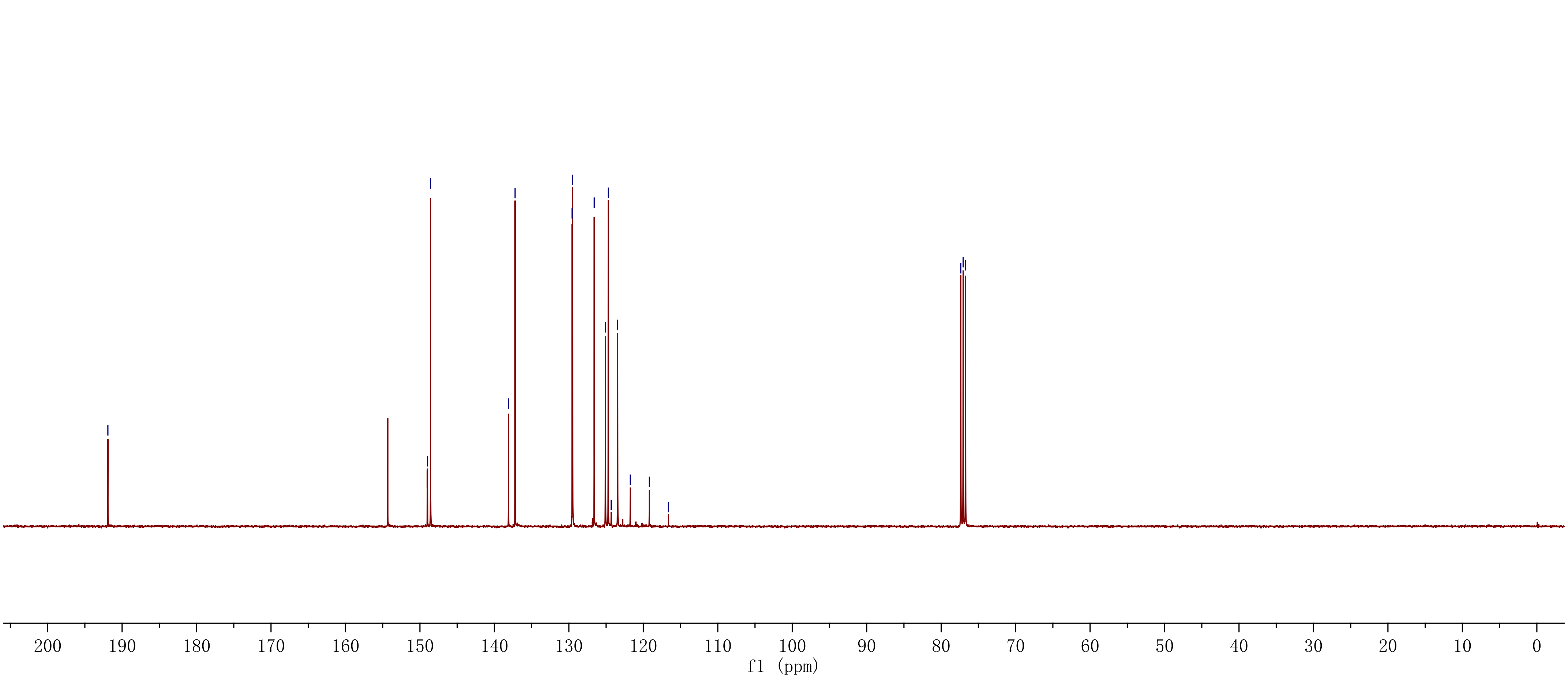

20231103zm
2e

—-57.8541

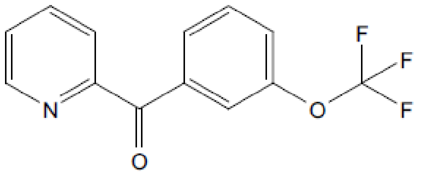

Figure S11. ^{19}F spectrum of **2e** in $CDCl_3$

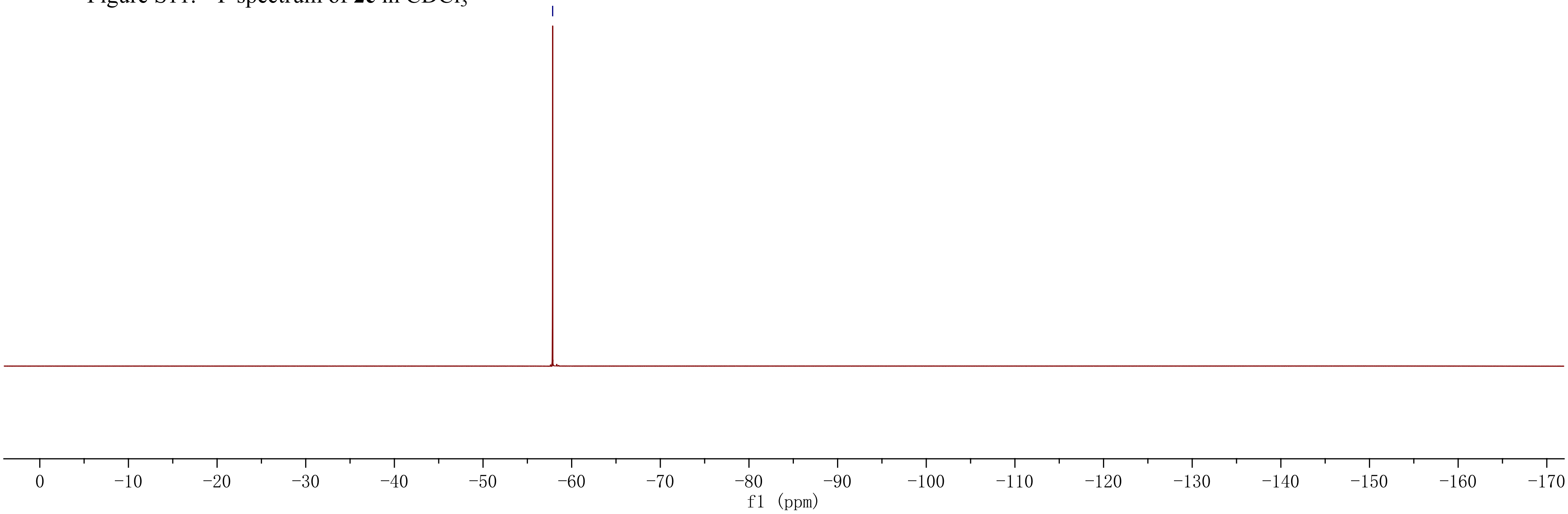

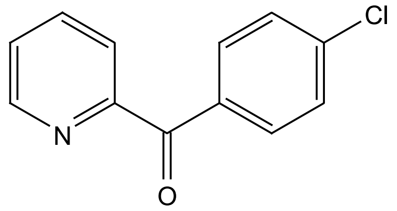

Figure S12. ^{1}H spectrum of **2f** in $CDCl_3$

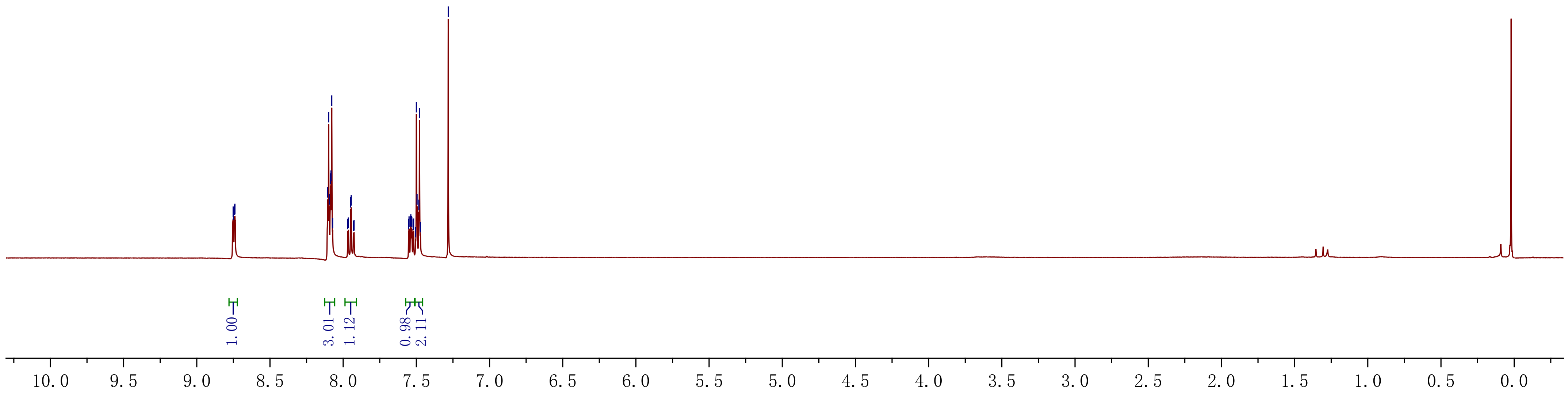

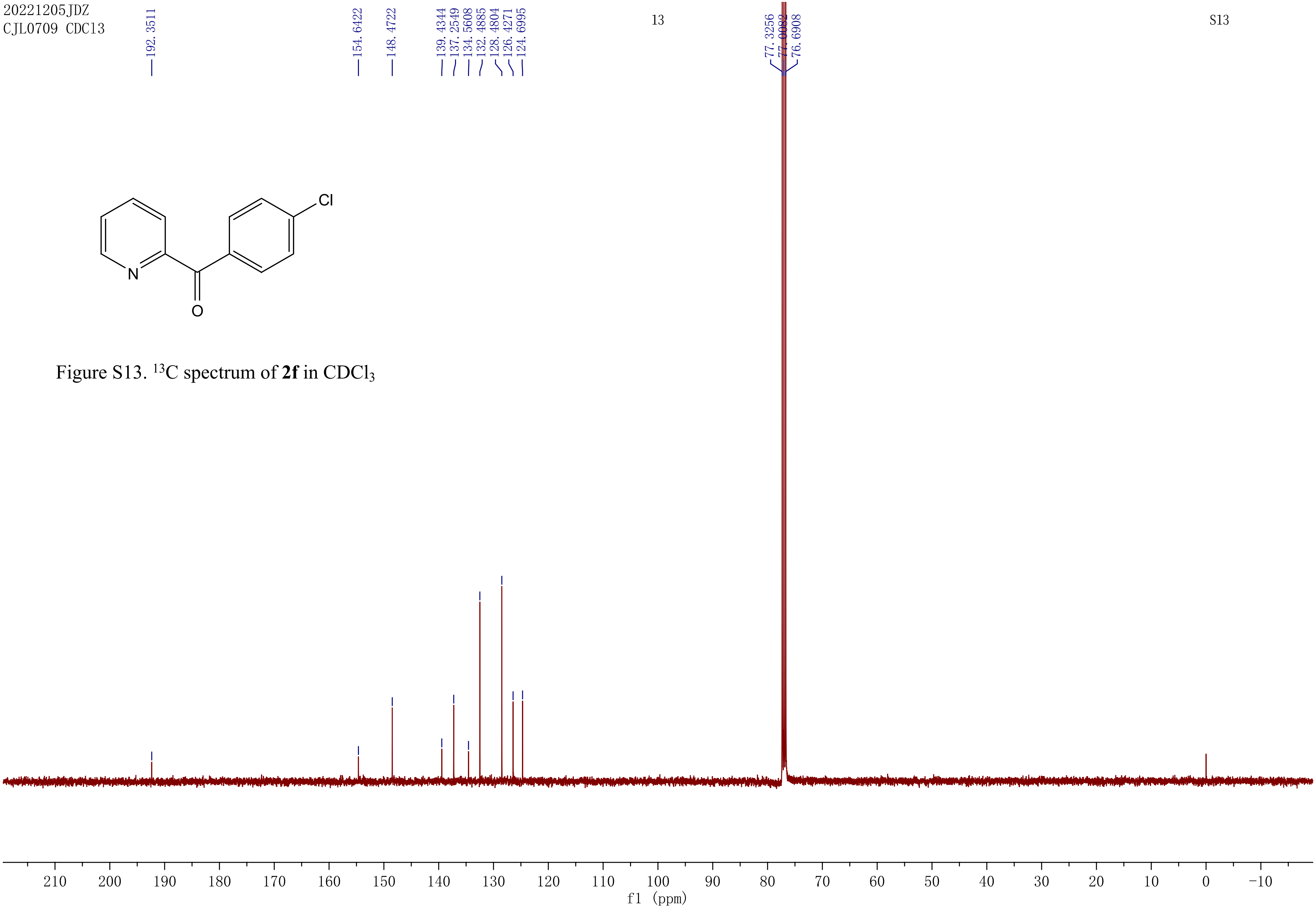

Figure S13. ^{13}C spectrum of **2f** in $CDCl_3$

20221025jdz
cjl0723

8.7636
8.7619
8.7598
8.7517
8.7500
8.1082
8.1048
8.1002
8.0881
7.9935
7.9468
7.5997
7.5971
7.5946
7.5919
7.5797
7.5771
7.5746
7.5719
7.5561
7.5532
7.5442
7.5412
7.5372
7.5342
7.5252
7.5223
7.4740
7.4544
7.4346
7.2819

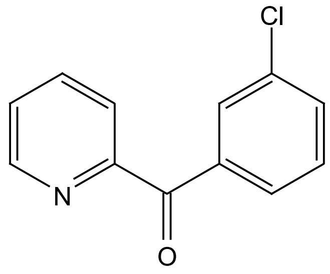

Figure S14. ^{1}H spectrum of **2g** in $CDCl_3$

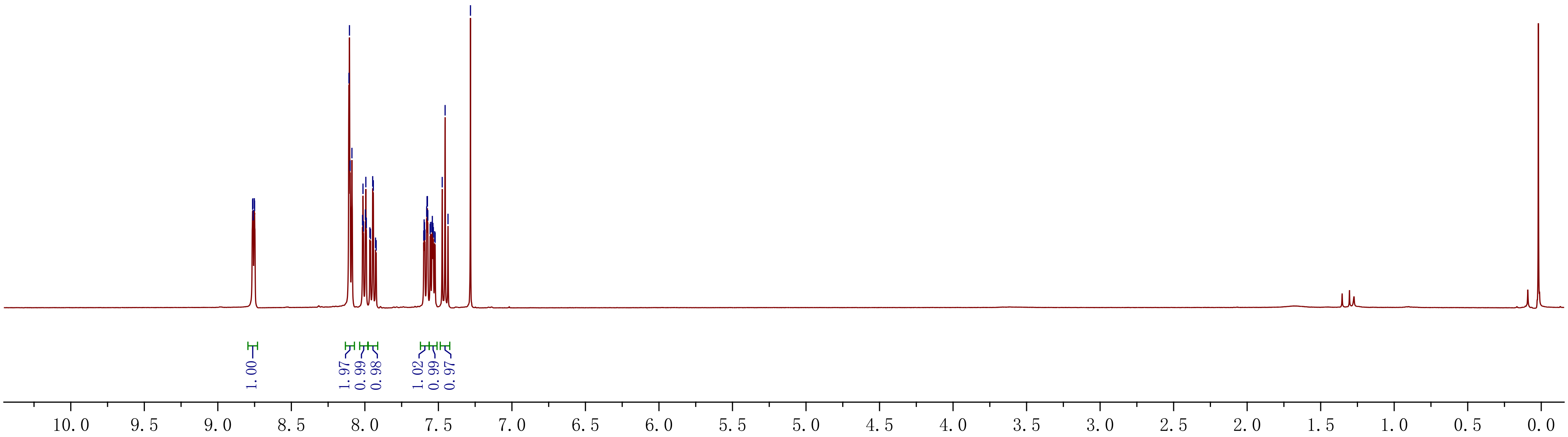

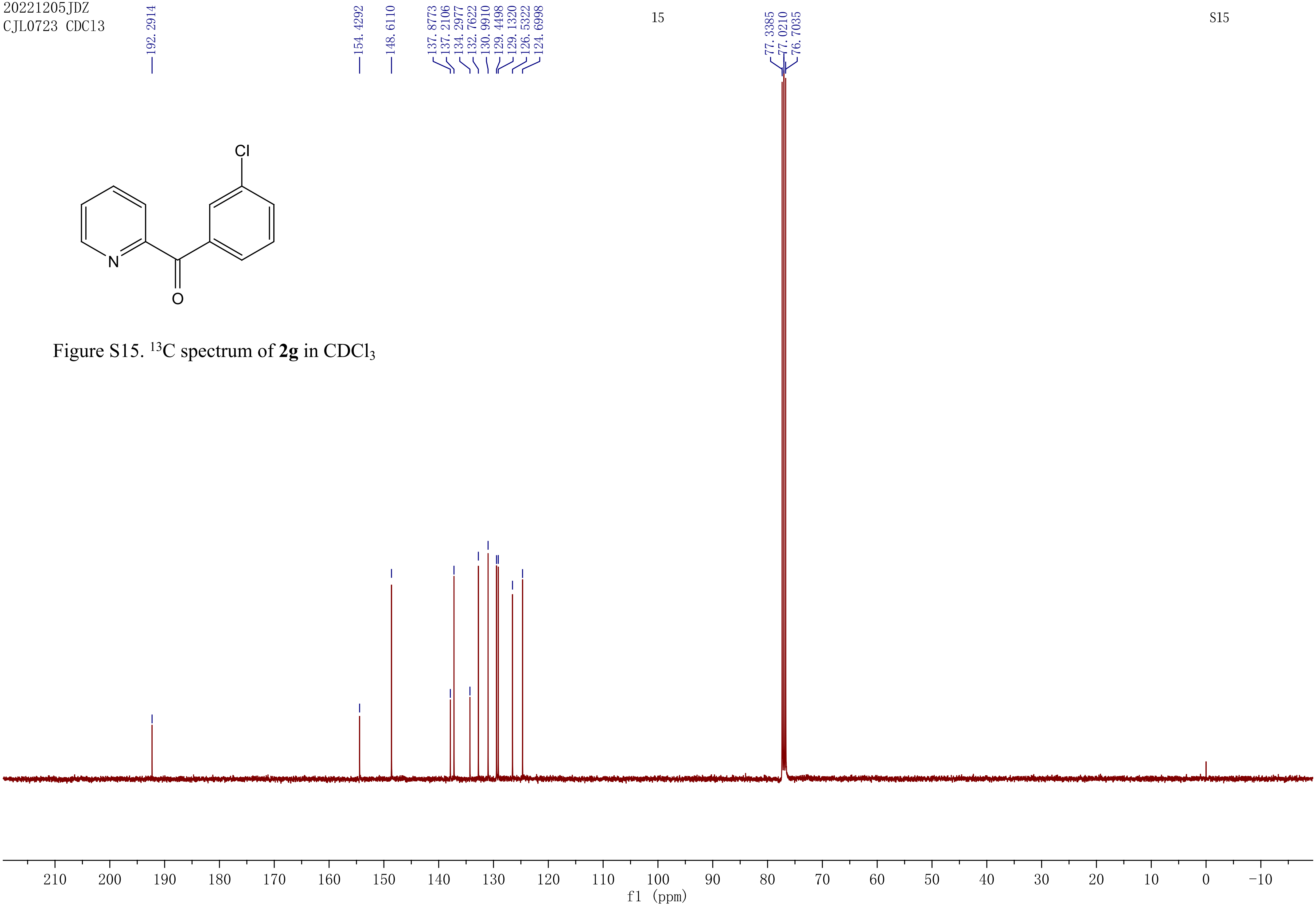

Figure S15. ^{13}C spectrum of **2g** in $CDCl_3$

CJL0805
CJL0805

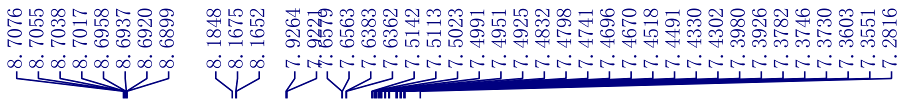

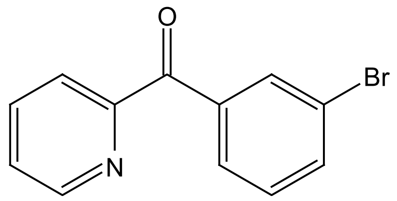

Figure S16. ^{1}H spectrum of **2h** in $CDCl_3$

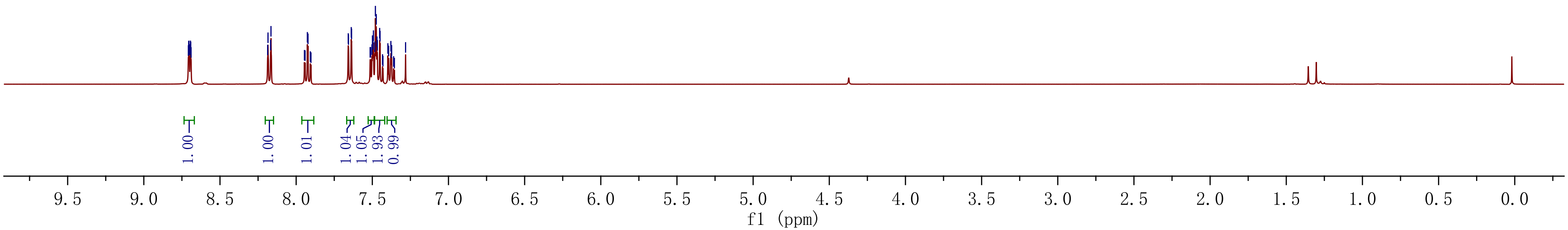

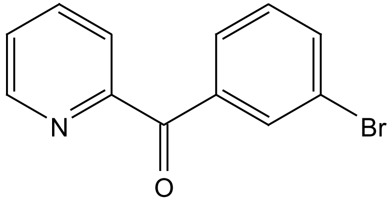

Figure S17. ^{13}C spectrum of **2h** in $CDCl_3$

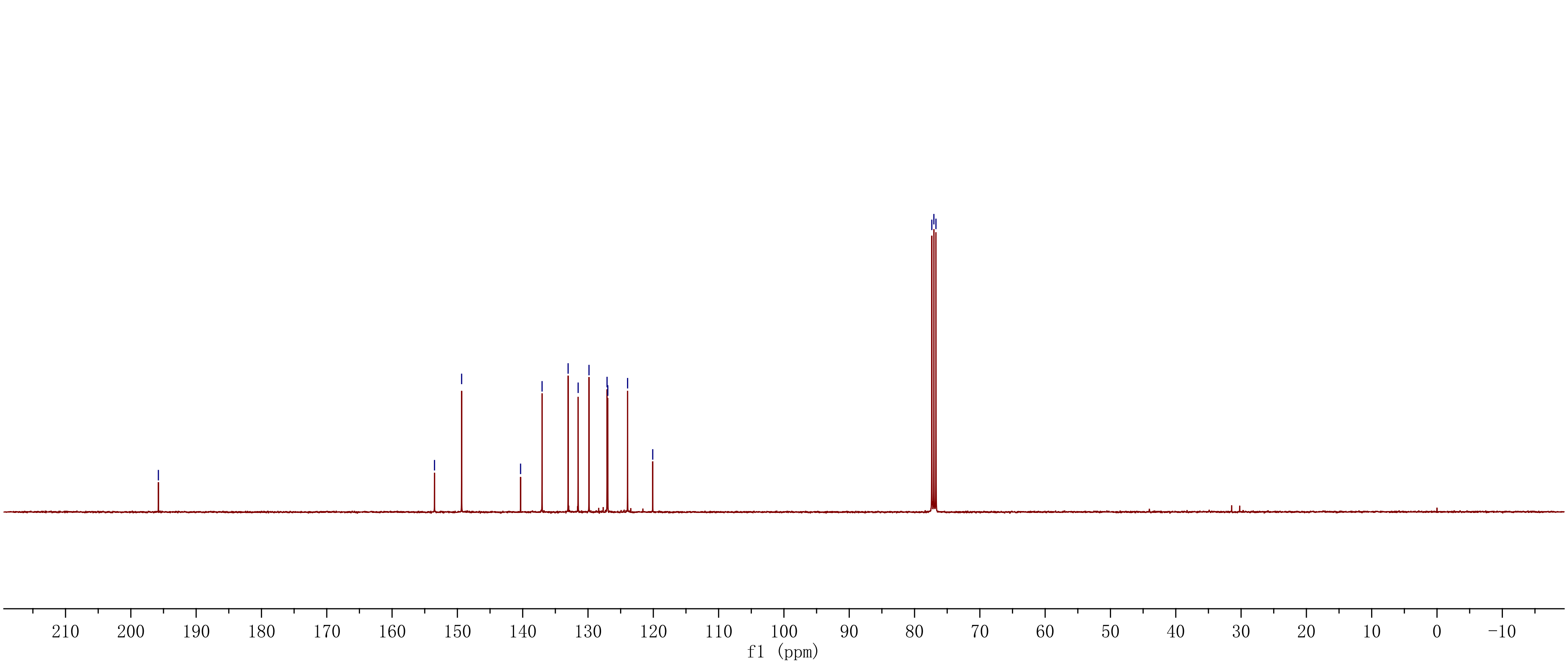

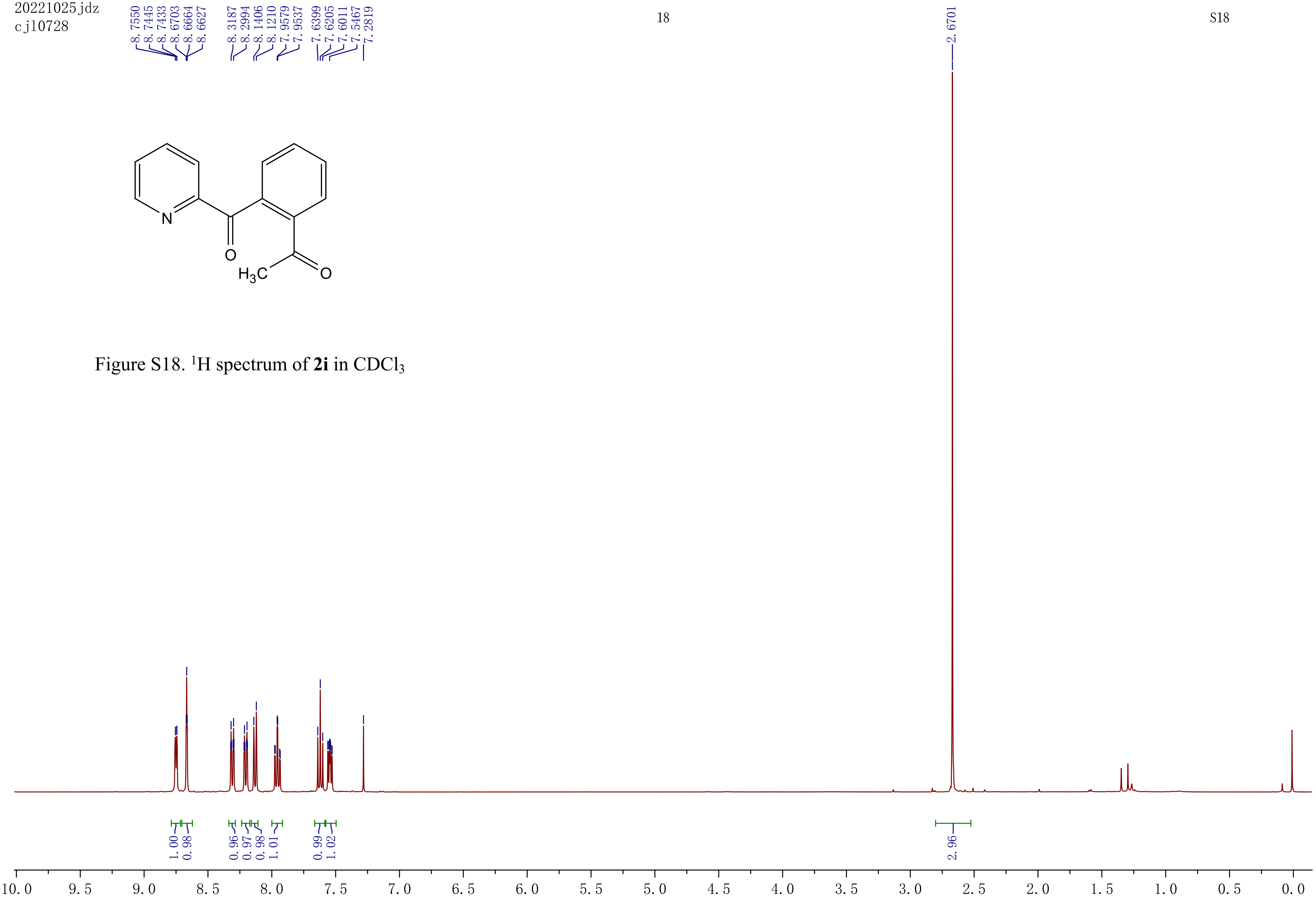

Figure S18. ^{1}H spectrum of **2i** in $CDCl_3$

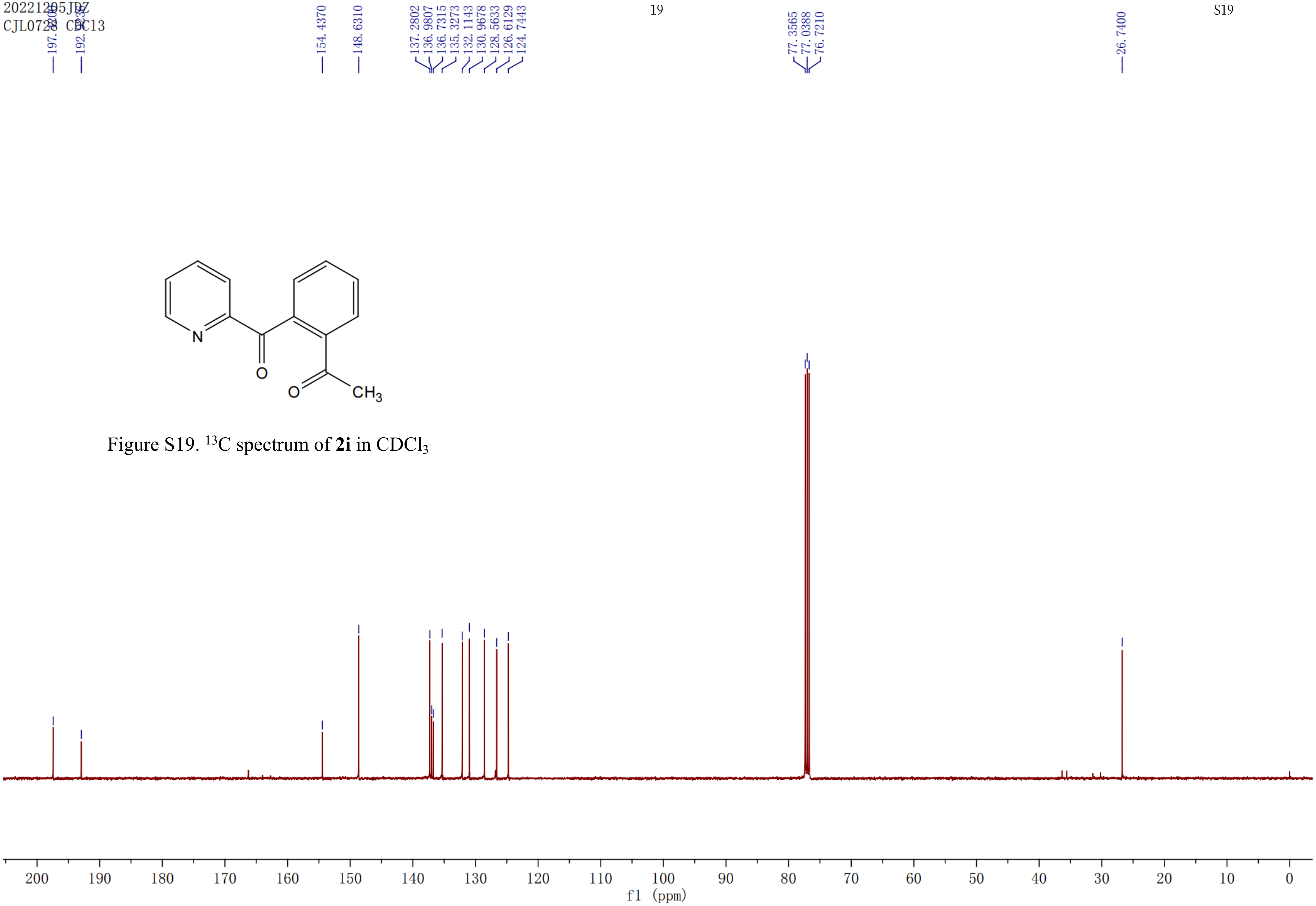

Figure S19. ^{13}C spectrum of **2i** in $CDCl_3$

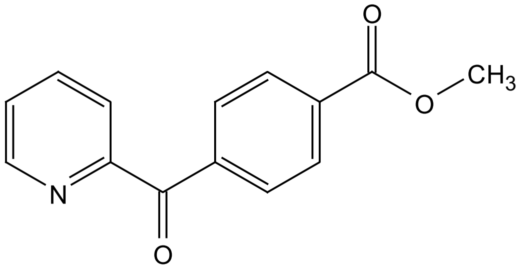

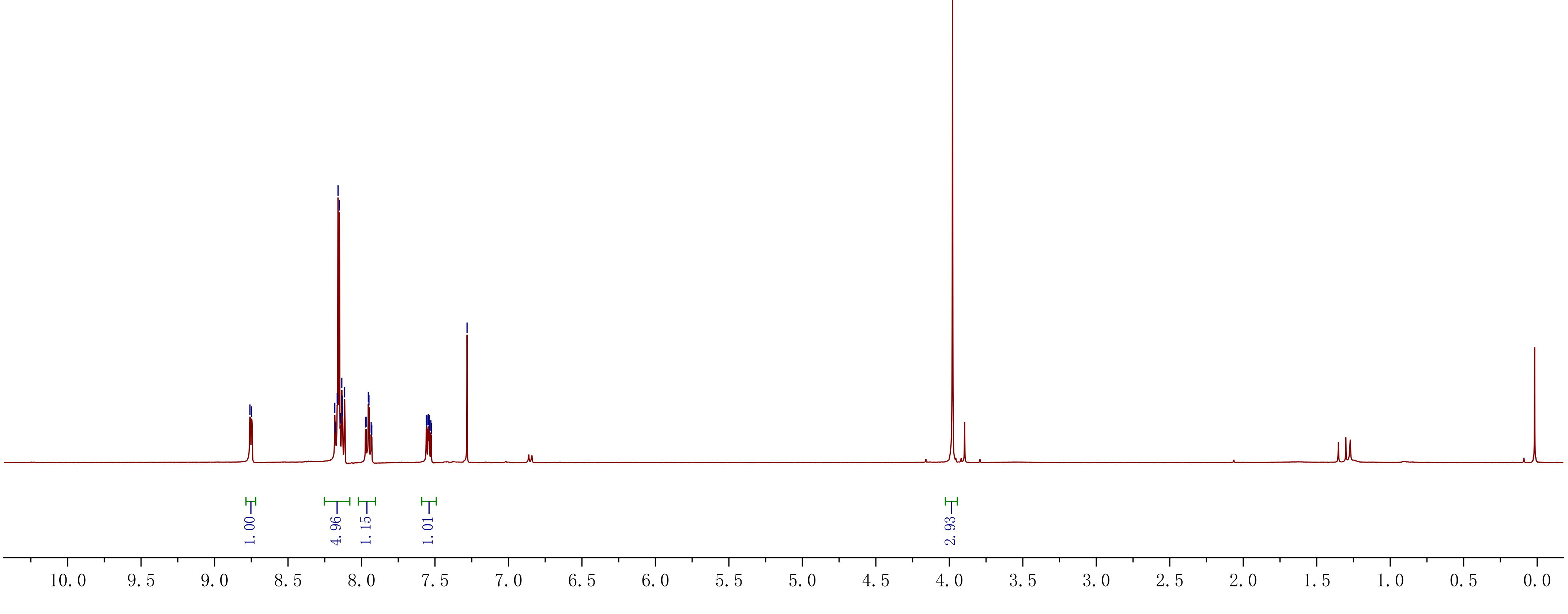

Figure S20. ^{1}H spectrum of **2j** in $CDCl_3$

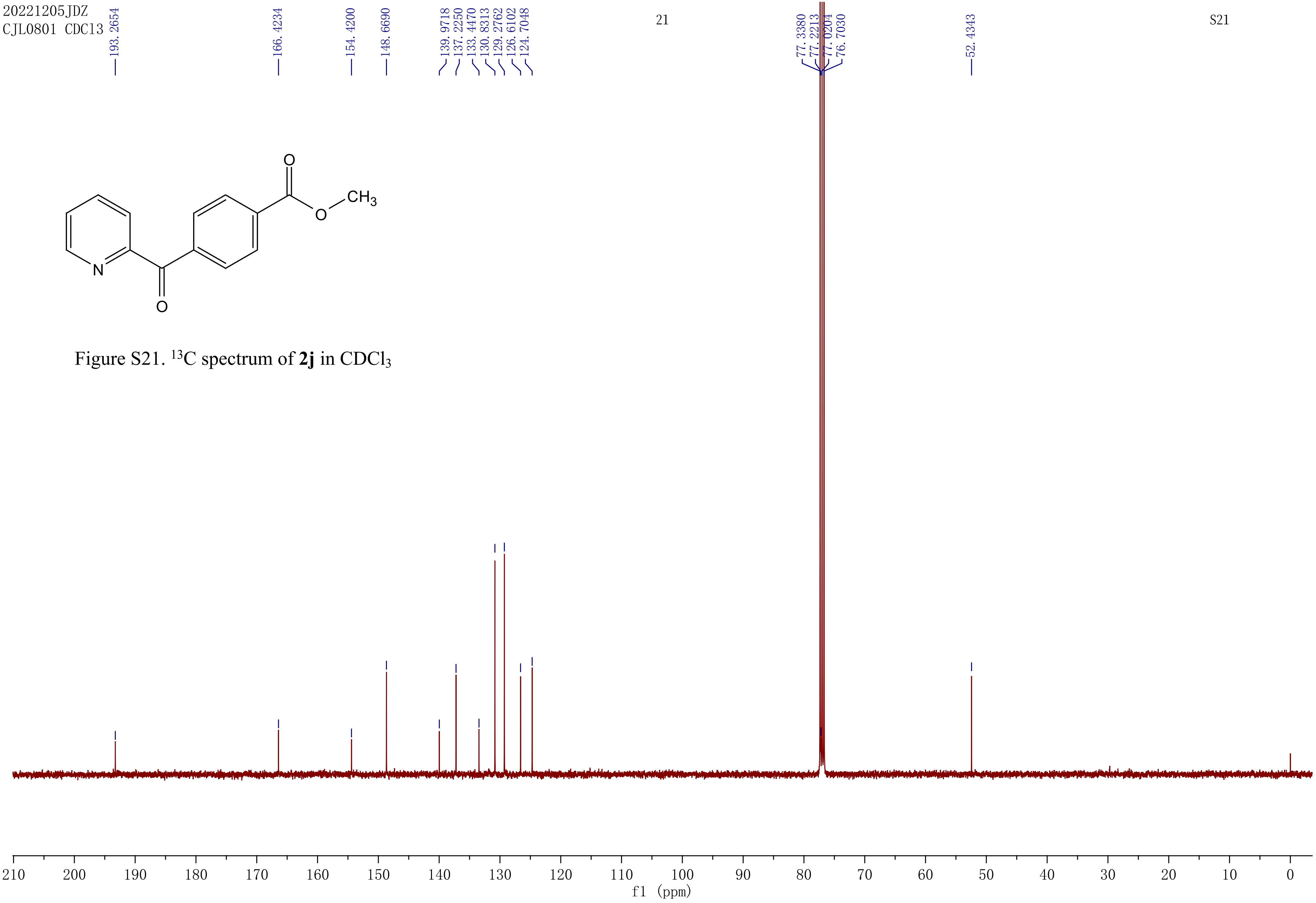

Figure S21. ^{13}C spectrum of **2j** in $CDCl_3$

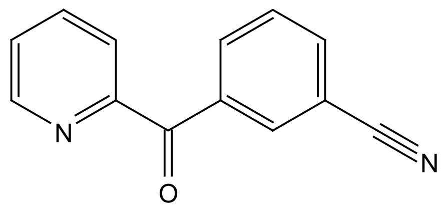

Figure S22. ^{1}H spectrum of **2k** in $CDCl_3$

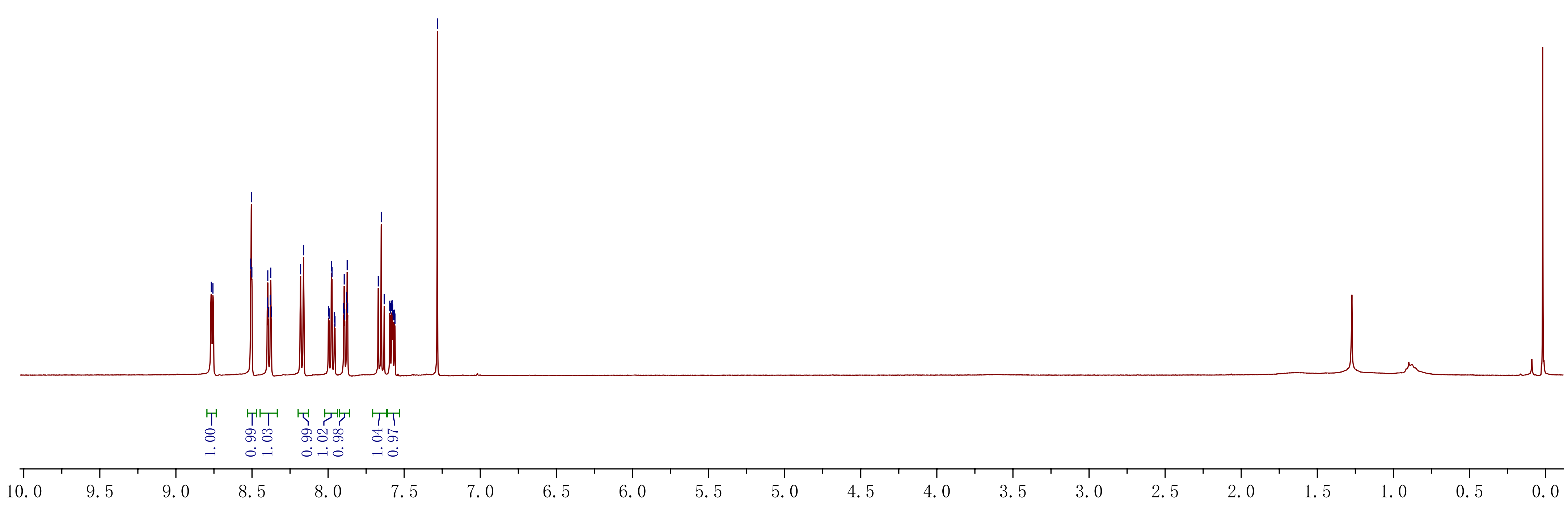

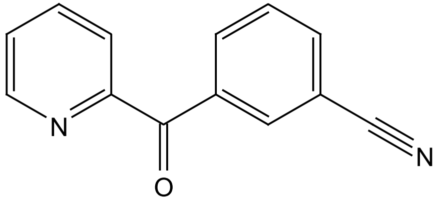

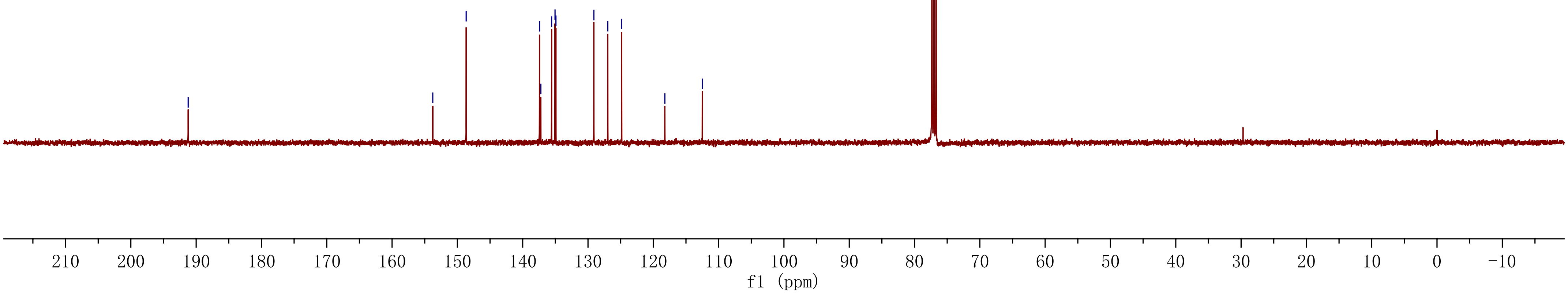

Figure S23. ^{13}C spectrum of **2k** in $CDCl_3$

20221025jdz
cjl0705

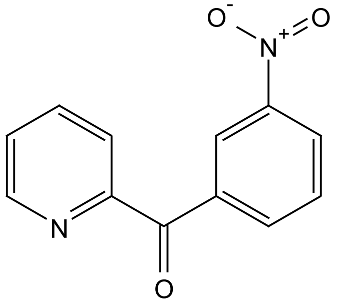

Figure S24. ^{1}H spectrum of **2l** in $CDCl_3$

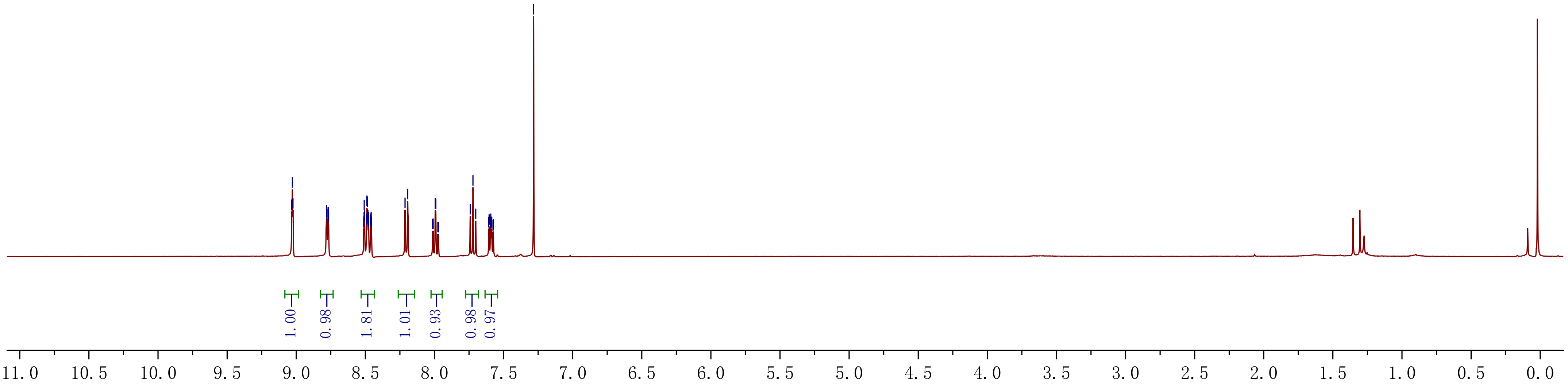

20221205JDZ
CJL0705 CDCl3

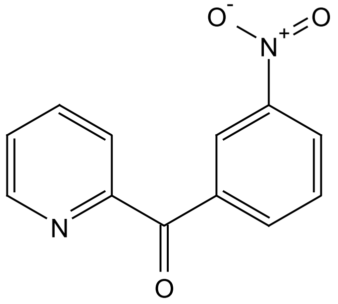

Figure S25. ^{13}C spectrum of **2l** in $CDCl_3$

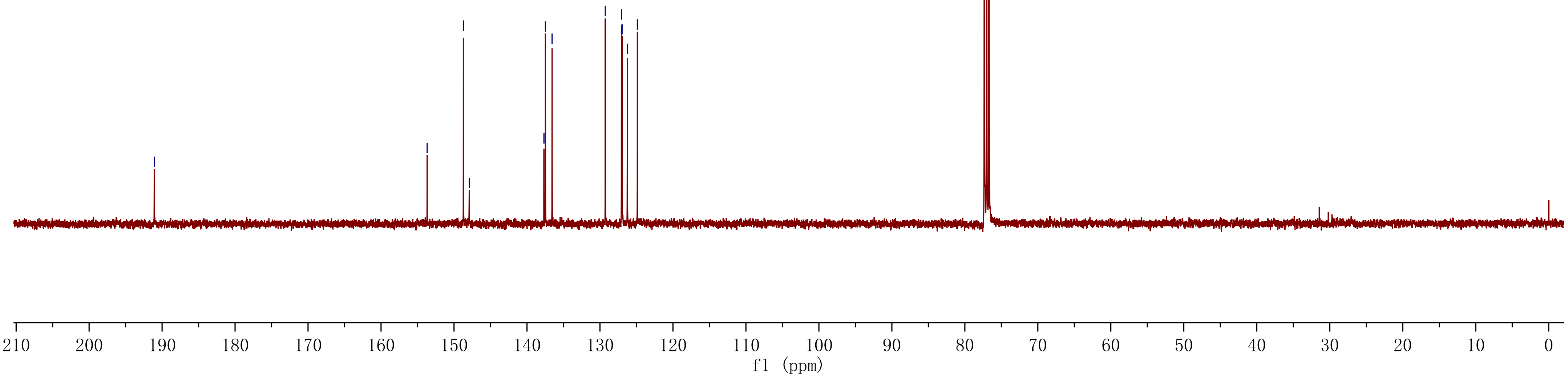

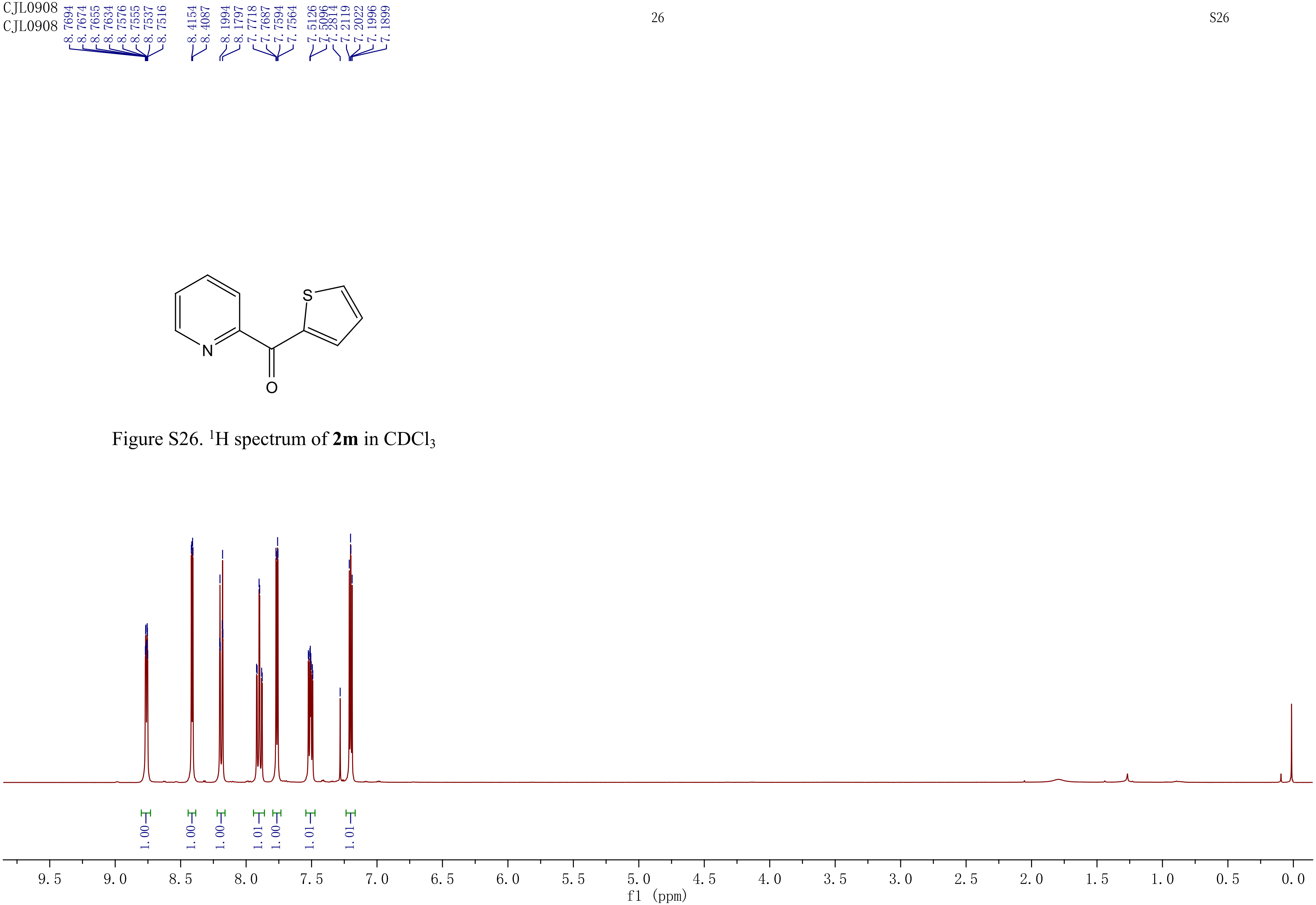

Figure S26. ^{1}H spectrum of **2m** in $CDCl_3$

20221209ZM
CJL0908 CDCl13

183.5436
154.0169
148.2598
140.0115
137.0986
136.6988
136.3153
127.5908
126.6786
123.7974
77.3668
77.0492
76.7315

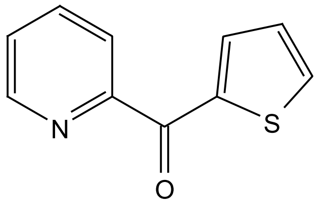

Figure S27. ^{13}C spectrum of **2m** in $CDCl_3$

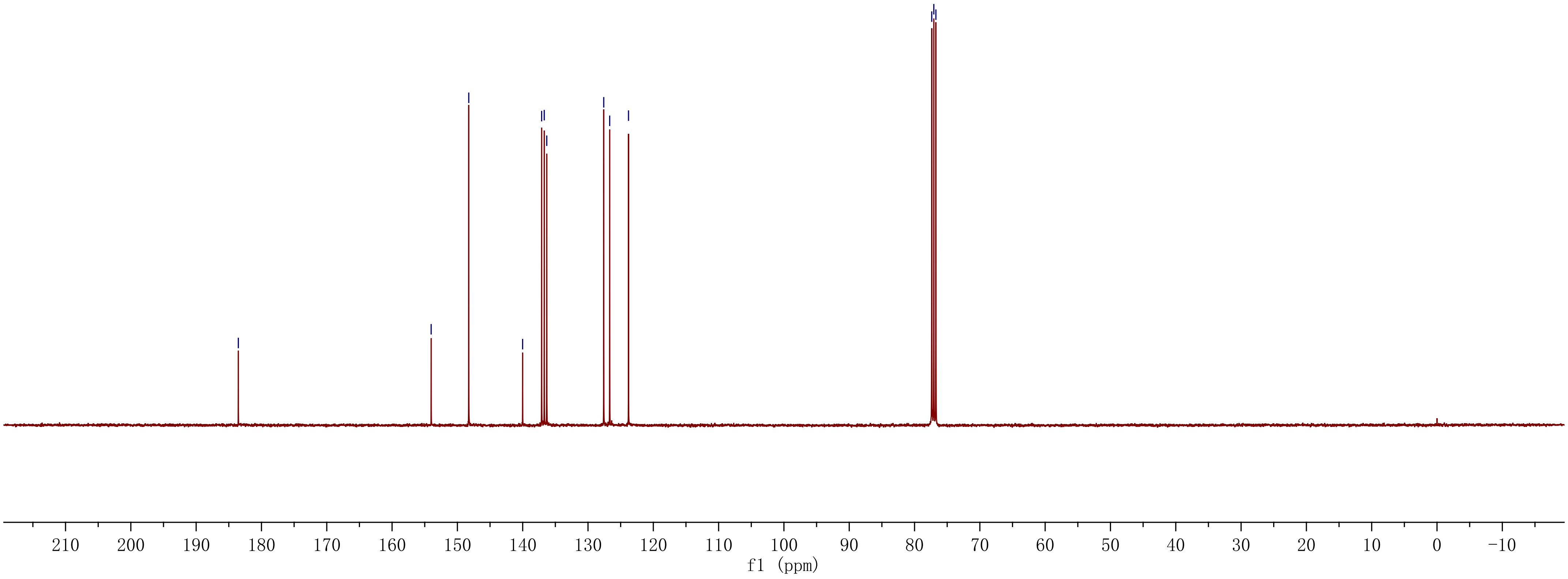

20221025jdz
cjl1002

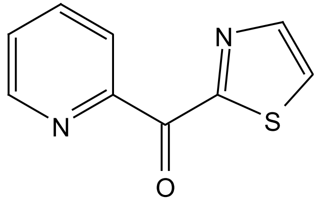

Figure S28. ^{1}H spectrum of **2n** in $CDCl_3$

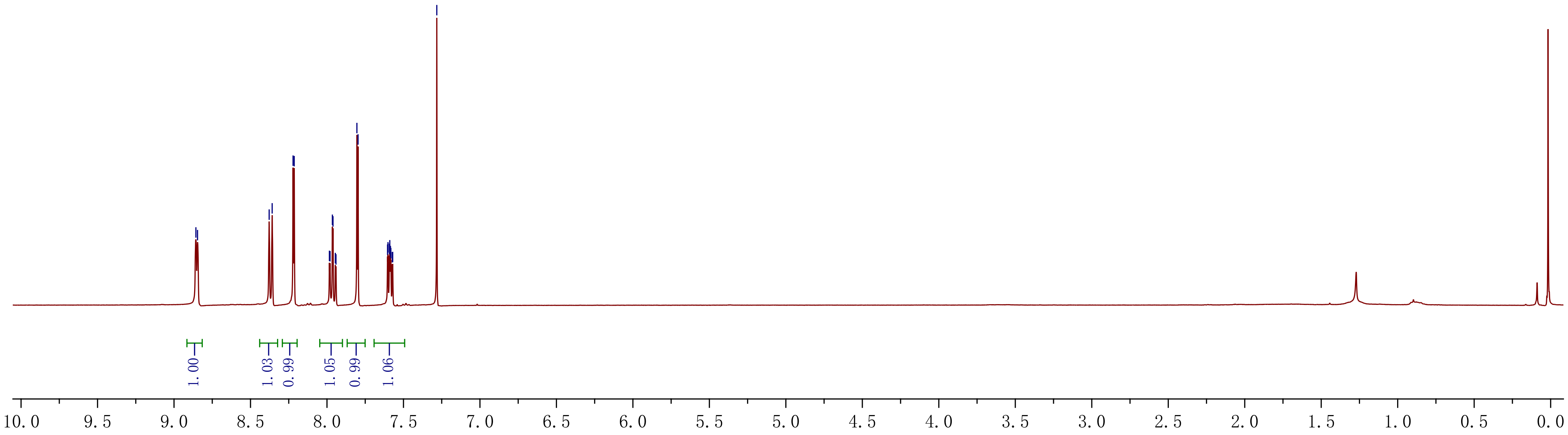

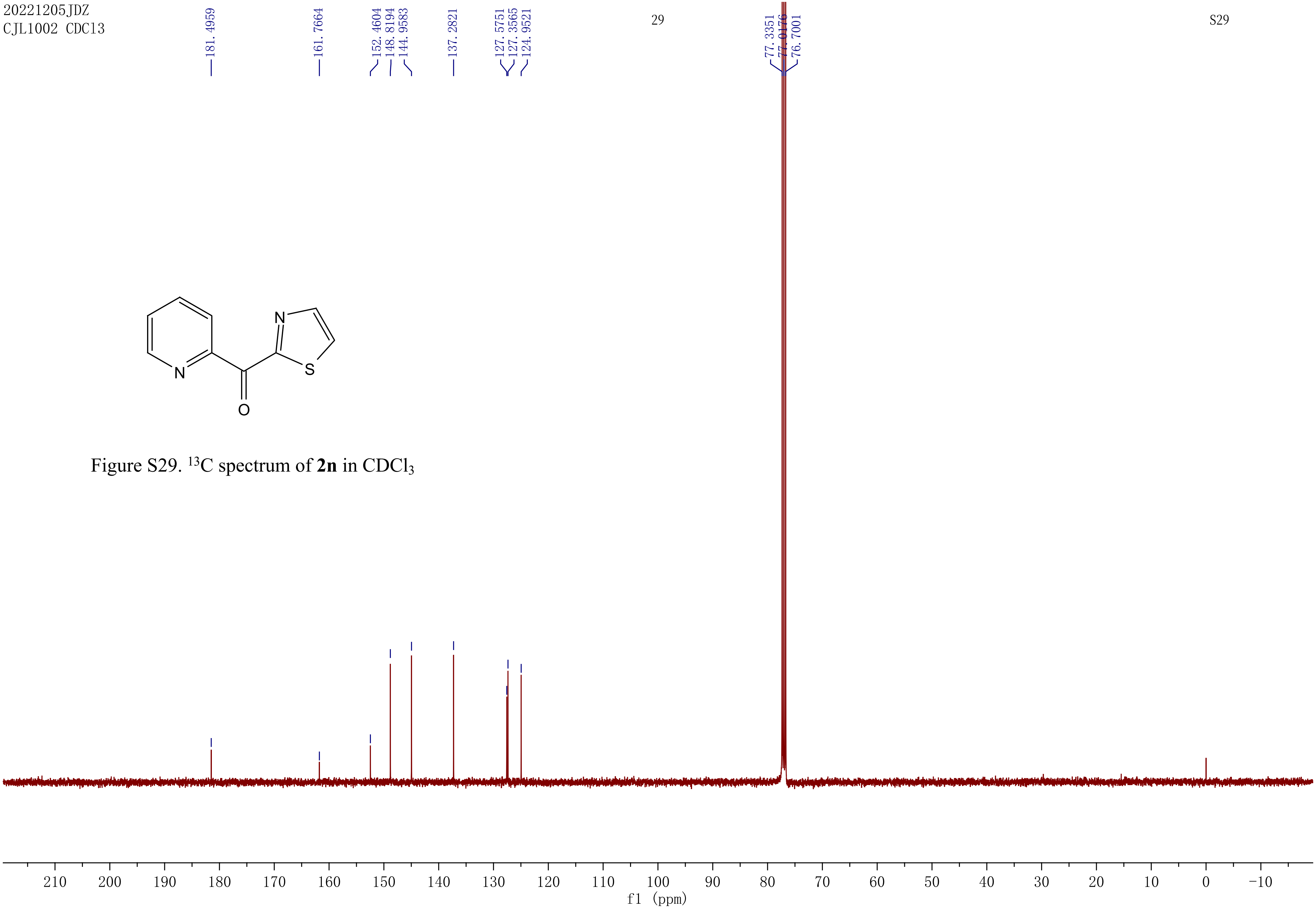

Figure S29. ^{13}C spectrum of **2n** in $CDCl_3$

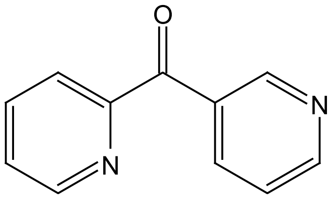

Figure S30. ^{1}H spectrum of **2o** in $CDCl_3$

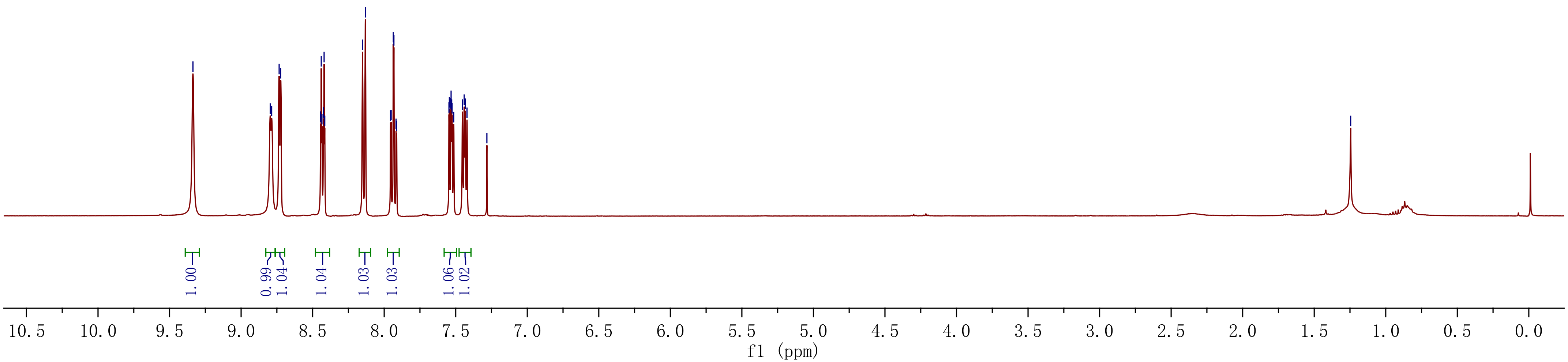

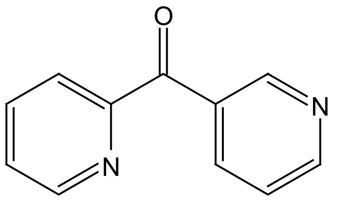

Figure S31. ^{1}H spectrum of **2o** in $CDCl_3$

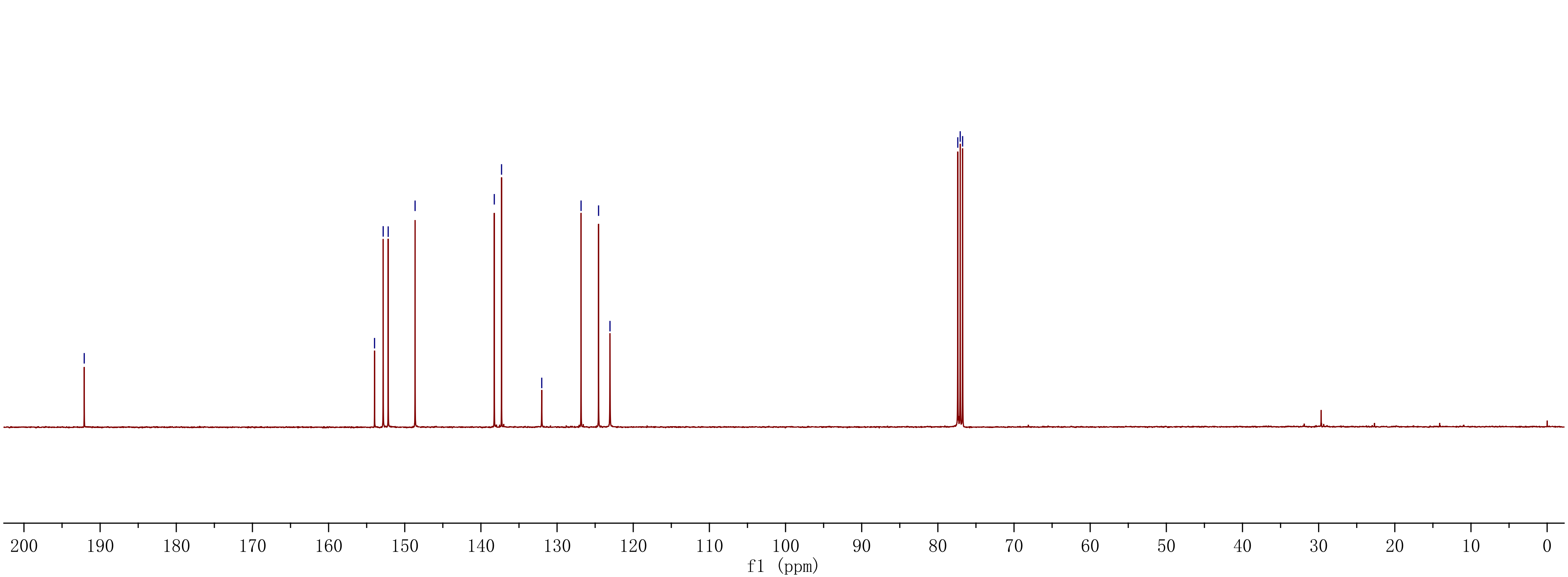

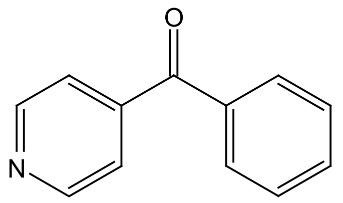

Figure S32. ^{1}H spectrum of **2p** in $CDCl_3$

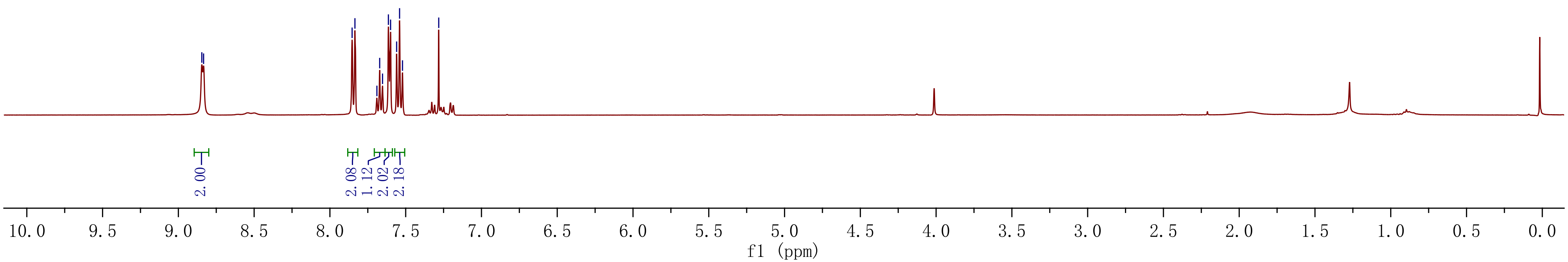

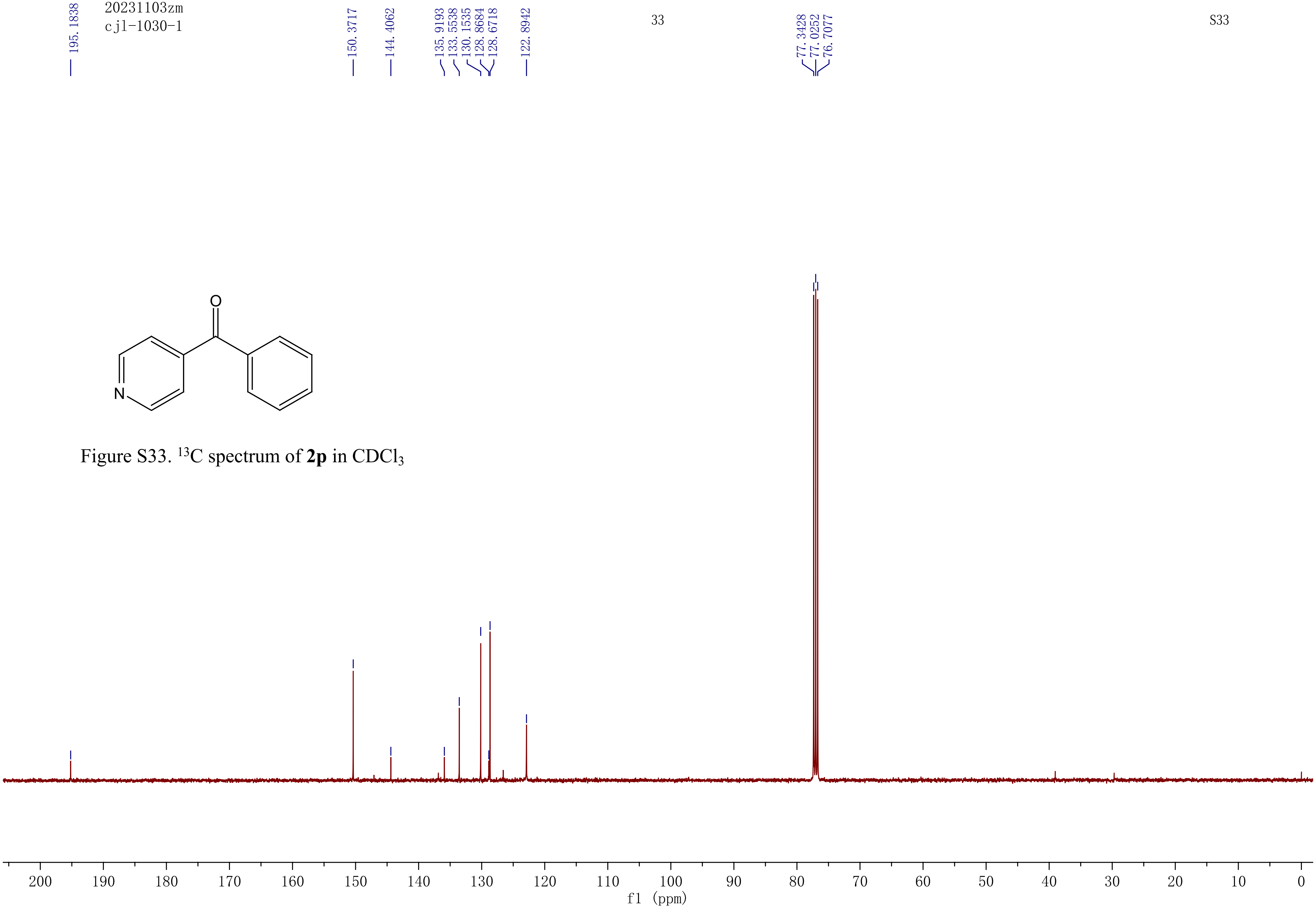

Figure S33. ^{13}C spectrum of **2p** in $CDCl_3$

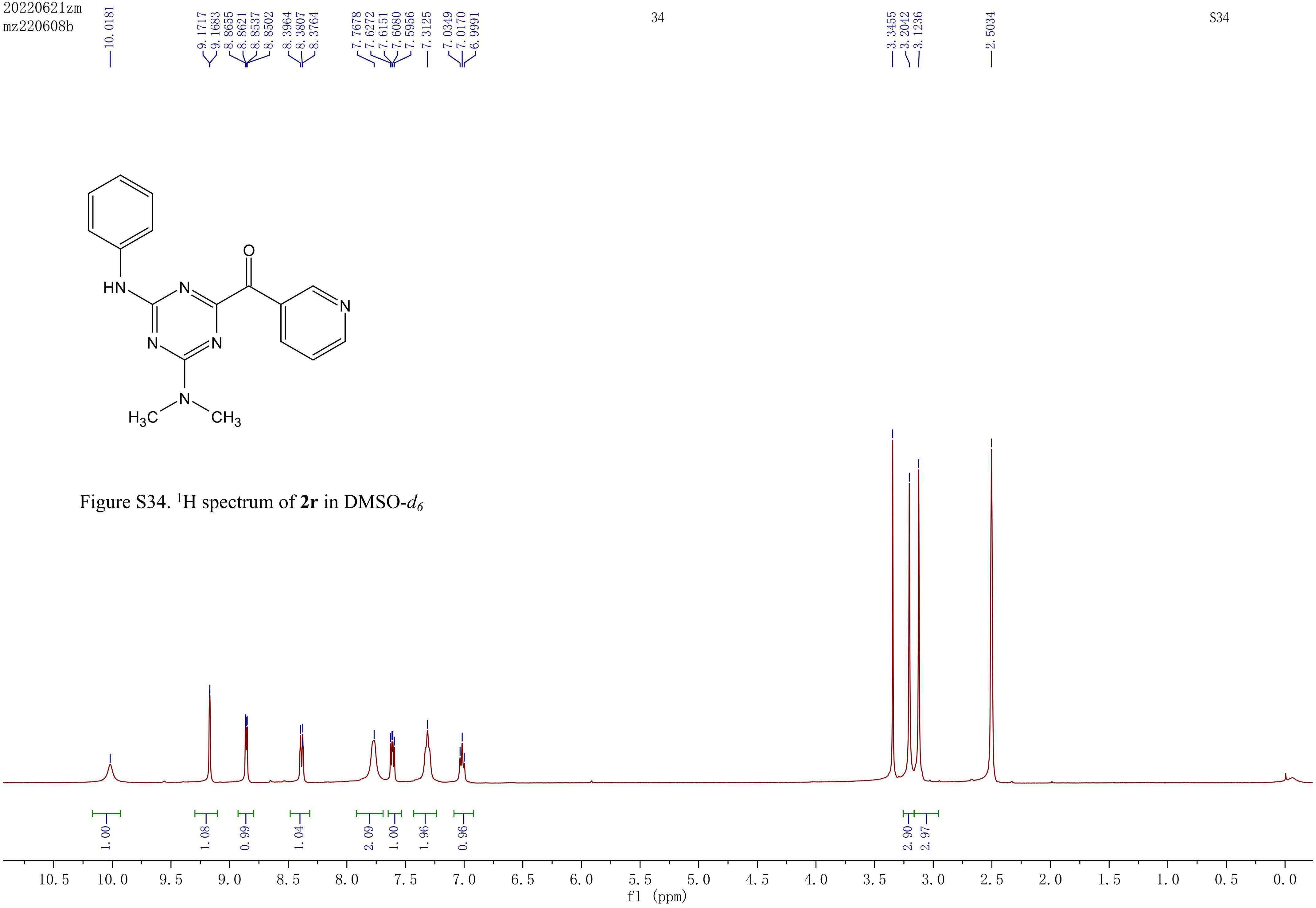

Figure S34. ^{1}H spectrum of **2r** in DMSO-d_6

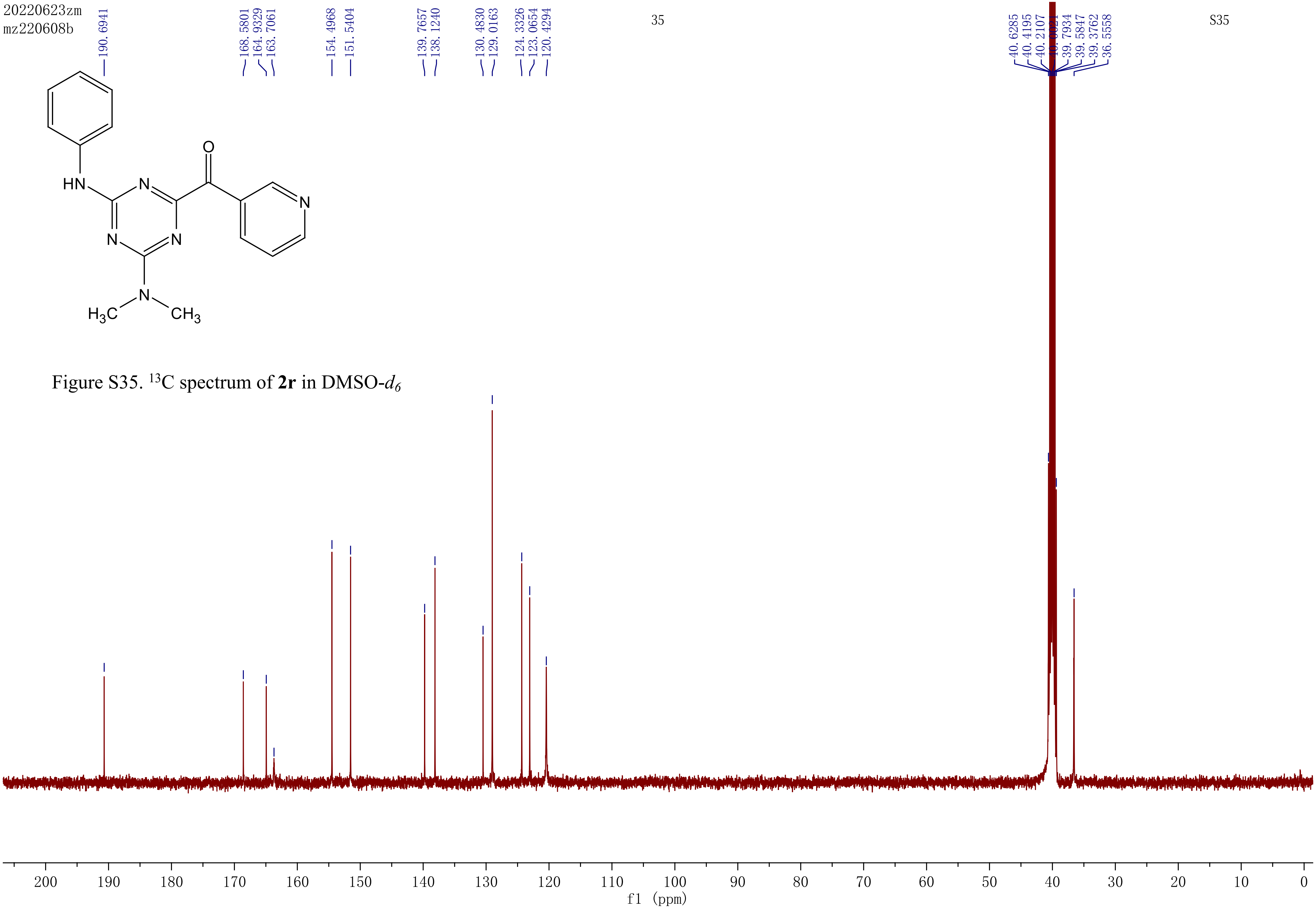

Figure S35. ^{13}C spectrum of **2r** in DMSO-d_6
